# A Global eDNA Comparison of Freshwater Bacterioplankton Assemblages Focusing on Large-River Floodplain Lakes of Brazil

**DOI:** 10.1007/s00248-016-0834-5

**Published:** 2016-09-09

**Authors:** Michael Tessler, Mercer R. Brugler, Rob DeSalle, Rebecca Hersch, Luiz Felipe M. Velho, Bianca T. Segovia, Fabio A. Lansac-Toha, Michael J. Lemke

**Affiliations:** 1 Sackler Institute for Comparative Genomics, American Museum of Natural History, Central Park W. at 79th St., New York, NY 10024 USA; 2 Richard Gilder Graduate School, American Museum of Natural History, Central Park W. at 79th St., New York, NY 10024 USA; 3 Biological Sciences Department, NYC College of Technology (CUNY), 300 Jay St., Brooklyn, NY 11201 USA; 4 Universidade Estadual de Maringá, Núcleo de Pesquisas em Limnologia, Ictiologia e Aquicultura - Nupelia, Av. Colombo, 5790 - Bloco G-90, Maringá, PR 87020-900 Brasil; 5 Biology Department, University of Illinois Springfield, One University Plaza, MS HSB223, Springfield, IL 62703 USA

**Keywords:** Bacterioplankton, Diversity, Floodplain lakes, Brazil, Metagenomics, Paraná, Pantanal Amazon, Araguaia

## Abstract

With its network of lotic and lentic habitats that shift during changes in seasonal connection, the tropical and subtropical large-river systems represent possibly the most dynamic of all aquatic environments. Pelagic water samples were collected from Brazilian floodplain lakes (total *n* = 58) in four flood-pulsed systems (Amazon [*n* = 21], Araguaia [*n* = 14], Paraná [*n* = 15], and Pantanal [*n* = 8]) in 2011–2012 and sequenced via 454 for bacterial environmental DNA using 16S amplicons; additional abiotic field and laboratory measurements were collected for the assayed lakes. We report here a global comparison of the bacterioplankton makeup of freshwater systems, focusing on a comparison of Brazilian lakes with similar freshwater systems across the globe. The results indicate a surprising similarity at higher taxonomic levels of the bacterioplankton in Brazilian freshwater with global sites. However, substantial novel diversity at the family level was also observed for the Brazilian freshwater systems. Brazilian freshwater bacterioplankton richness was relatively average globally. Ordination results indicate that Brazilian bacterioplankton composition is unique from other areas of the globe. Using Brazil-only ordinations, floodplain system differentiation most strongly correlated with dissolved oxygen, pH, and phosphate. Our data on Brazilian freshwater systems in combination with analysis of a collection of freshwater environmental samples from across the globe offers the first regional picture of bacterioplankton diversity in these important freshwater systems.

**Electronic supplementary material:**

The online version of this article (doi:10.1007/s00248-016-0834-5) contains supplementary material, which is available to authorized users.

## Introduction

Tropical lakes differ in a number of respects from temperate lakes in factors affecting turnover and biogeochemical cycles that have strong effects on species composition [17, 19, 31, 48]. Both local and regional processes can influence biodiversity at various spatial scales with the biodiversity of many remote places still being relatively unknown [9, 10].

Floodplains associated with major rivers have regular flood pulses where water level increases significantly for a period of time and then returns to baseline flow [27]. Fluvial dynamics and temperature are thought to be the main ecological driving force that acts on the communities present in these ecosystems (in [57]). Studies have shown that floodplains have high biodiversity and floodplain lakes are of fundamental importance in maintaining populations of species (e.g., [1, 52, 59]). Flood pulses help structure aquatic communities in floodplains at many levels, including the benthic community, phytoplankton, protozooplankton, zooplankton, fish, and aquatic macrophytes [2, 25, 27, 29, 41, 45, 49, 56, 58].

The Amazon River is one of the major biomes on the planet and is thought to support one third of all living species. The 7 million km^2^ Amazon basin is the largest watershed on Earth and contributes 12 % of all surface water that enters the ocean. The Paraná River holds the last stretch of (Brazilian) undammed river and several conservation units including the Ilha Grande National Park, the State Park of Ivinheima River, and the islands and wetlands of the Paraná River environmental protection area, currently being assessed for inclusion as a Biosphere Reserve by UNESCO. One area of the Pantanal, the largest continuous wetland on the planet, has a unique biome with high biological productivity that has qualified two of its wetland areas as international UNESCO Biosphere Reserves and World Heritage Sites. Despite evidence of increased human impacts [22, 53], few limnological studies have been conducted in this region. The Araguaia River basin has about 76 % of the drainage area covered by the Cerrado, one of 25 hotspots of biodiversity in Brazil [36], and includes a transition region of the Amazon rainforest.

Despite past efforts to determine the biodiversity of Brazilian aquatic systems, a representative part of this overall biodiversity remains unknown with information on the smaller communities, especially bacterioplankton communities, being particularly deficient. To shed light on the diversity in these freshwater systems, we addressed the following objectives: (1) to determine how the bacterioplankton composition in Brazilian floodplain freshwater systems compare in a global context to other freshwater systems and (2) to expand knowledge on the biodiversity and distribution of bacterioplankton in the four great river-floodplain ecosystems in Brazil.

## Materials and Methods

### Study Sites, Sampling, and Field Measurements

**Fig. 1:**
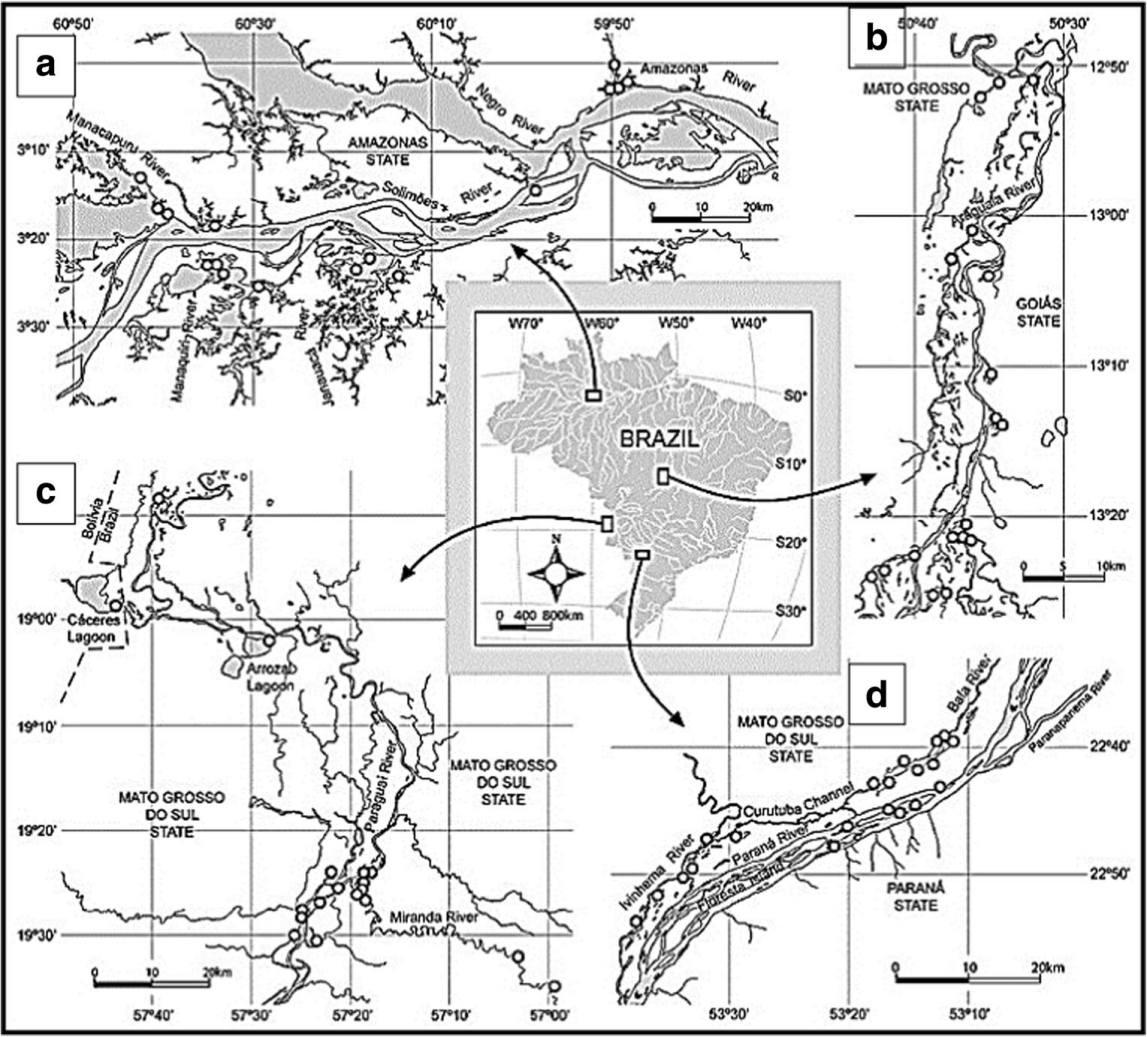
Reaches of the four Brazilian rivers featured in this study indicating floodplain lake sampling sites: Amazon (**a**), Araguaia (**b**), Pantanal (**c**), and Parana (**d**)

Samples were taken in river floodplain lakes from the Amazon, Araguaia, Pantanal region (Paraguai and Miranda Rivers), and Paraná Rivers (Fig. 1). Lakes sampled, date of sampling, and river association are shown in Supplemental Table 1. Water (∼5 cm below surface) was collected and filtered in the field through either Sterivex filters using a syringe or through 0.2 μm Isopore membranes (Millipore, Billerica, MA), refrigerated in the field, and then frozen until processed. Dissolved oxygen and temperature (Oxymeter YSI 550A), conductivity (Digimed DM-3P), turbidity (Motte 202VE), and water transparency (Secchi disk) were measured in the field. Nutrients (i.e., soluble nitrogen and phosphate) were measured in the laboratory as described in Lemke et al. [30].

### Molecular Analysis

Environmental DNA (eDNA) was extracted with FastDNA kits (MP Biomedicals, Solon, OH) and quantified using the dsDNA High Sensitivity Assay kit on the Qubit® 2.0 Fluorometer (Invitrogen, Waltham, MA). All samples with DNA concentration over 0.6 ng/μL were amplified in 25 μL reactions using either a FastStart High-Fidelity PCR System (Roche) or Q5® High-Fidelity DNA Polymerase (New England BioLabs) and HPLC-purified fusion primers targeting the 16S rRNA gene. Details on the molecular biology techniques such as amplification primers, amplification conditions, and DNA sequencing are as follows: Primer B-341F (5′-CCT ATC CCC TGT GTG CCT TGG CAG TCT CAG CCT ACG GG NGG CWG CAG-3′) did not contain a multiplex identifier (MID); rather, it only included the emPCR and sequencing primer (CCTATCCCCTGTGTGCCTTGGCAGTC), key tag for amplicon sequencing (TCAG), and the 16S primer (CCTACGGGNGGCWGCAG; [21]). We utilized 12 MID-806R primers (5′-CCATCTCATCCCTGCGTGTCTCCGACTCAGACGAGTGCGTGGACTACHVGGGTWTCTAAT-3′; CCATCTCATCCCTGCGTGTCTCCGAC hybridizes to Lib-L capture bead, TCAG is the key tag, and GGACTACHVGGGTWTCTAAT is the 16S primer [earth microbiome project]), each of which had a different 10-bp MID adaptor (position denoted by ACGAGTGCGT). We amplified each sample in triplicate to mitigate reaction-level PCR biases. Cycling parameters were similar to those presented in Bates et al. [5]: initial denaturation 95 °C, 3 min; 35 cycles (95 °C, 30 s; 57 °C, 35 s; 72 °C, 55 s); final extension 72 °C, 7 min (template concentration 5–10 ng/μL). Prior to PCR cleanup, triplicate PCR reactions for each sample were pooled. Successful amplifications (58 samples) were checked for length on a 2100 Bioanalyzer using a DNA 7500 kit (Agilent Technologies) and cleaned twice with Agencourt AMPure XP (Beckman Coulter) to remove remnant primer dimer and small fragments. Both cleanings followed Roche procedures with two adjustments: AMPure was not eluted in sizing solution and 1.2× AMPure concentration was used for the second iteration. Cleaned amplicons were quantified on the QuantiFluor ST Fluorometer (Promega) and diluted to 1 × 10^9^ molecules/μL in 1× TE buffer. We pooled 12 MID-labeled PCR products into a single tube. Pooled amplicons were diluted to 1 × 10^7^ molecules/μL in molecular grade water. Emulsion-based clonal amplification, bead washes and recovery, DNA library bead enrichment, and sequence primer annealing were carried out using the GS Junior Titanium emPCR (Lib-L) Kit following the manufacturer’s protocols as outlined in the *emPCR Amplification Method Manual* (*Lib-L*) (v. April 2011). Enriched beads were prepared for sequencing on a GS Junior PicoTiterPlate Device using the GS Junior Titanium Sequencing Kit and following the manufacturer’s protocols as outlined in the *Sequencing Method Manual* (v. November 2011). Single-end massively parallel pyrosequencing was carried out in multiplex on a 454 GS at the Sackler Institute for Comparative Genomics, American Museum of Natural History, New York, NY, USA. Postsequencing processing involved a multitiered approach to assure the quality of downstream sequence data, and began by demultiplexing the data and implementing five standard 454 quality filters on the GS Junior (Dot, Mixed, Signal Intensity, Primer and TrimBack Valley). Thereafter, sff_extract (http://bioinf.comav.upv.es/sff_extract/index.html) was used to create .fasta, .fasta.qual, .fastq, and .xml files. In addition, sff_extract clipped key/adaptor sequences and removed low-quality reads (i.e., any base listed in lower case). After visualizing the results of sff_extract using FastQC (http://www.bioinformatics.babraham.ac.uk/projects/fastqc/), two binaries, FASTX_trimmer and FASTQ_quality_trimmer, both part of the FASTX toolkit (http://hannonlab.cshl.edu/fastx_toolkit/), were used to further trim low-quality regions; only bases with a Phred quality score ≥25 were retained in the final dataset. After utilizing FASTX_trimmer and FASTQ_quality_trimmer, FastQC was again used to visualize and verify the overall quality of the reads. The data have been deposited with links to BioProject accession number PRJNA310230 in the NCBI BioProject database (https://www.ncbi.nlm.nih.gov/bioproject/).

### Global Comparisons

The Short Read Archives (SRA) at NIH/NCBI were used to obtain as many freshwater samples from diverse locations around the globe as possible as of November, 2015. Our criteria for inclusion of data into this study was first that the dataset had to be available in the SRA, and second, at least five samples per location were preferred for inclusion (Supplemental Table 2). We did include one smaller study (Lake Ladoga [*n* = 3]) because it filled in a geographic gap. The sequence information from the South African sites in the analysis were taken from freshwater and sediment samples. In this case, we retained these samples in the study because they were the only African samples in the short read archives. We categorized the data into broad geographical units: Europe, southern Africa, Asia, North America, and South America. The SRA files were converted to fastq files using *fastq-dump.2.4.3* (http://www.ncbi.nlm.nih.gov/Traces/sra/), and the fasta files from our Brazil study were then uploaded to the MG-RAST website [35] where rarefaction curves were generated (Supplemental Fig. 1). Next, we used the RDP Classifier (http://rdp.cme.msu.edu/classifier/classifier.jsp) to classify the sequences by sites at the phylum and family levels for each of the global sites. Each sample from the various geographic units including Brazil was then compared as outlined below.

### Data Analysis and Diversity

**Fig. 2:**
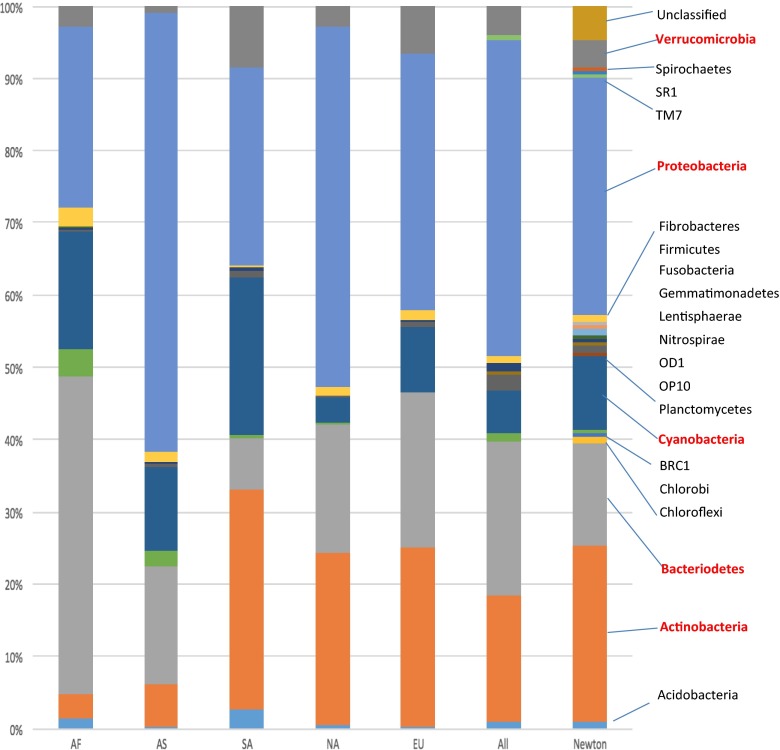
*Bar graphs* comparing the overall results of the Newton et al. [38] meta-analysis of lake systems based on amplicon-cloned Sanger-generated sequences at the phylum level. In that study, the authors were able to compare the diversity patterns using “full length” 16S rDNA sequences and for shorter sequences. We show comparison to our metadataset for the shorter sequences (<1300 bp). Methods for construction of the graph are given in the text. The continental abbreviations are as follows: *NA* North America, *SA* South America, *EU* Europe, *AS* Asia, *AF* Africa

Diversity at the family and phylum levels was assessed by comparing classifications found by the RDP categorizer. We used this approach to assess both broad (phylum) and narrow (family) levels of taxonomic diversity. Lists of taxonomic assignment for each sequence in each dataset were compiled and used for comparisons of taxon richness, nonmetric multidimensional scaling analyses (NMDS) and comparison of identified and unidentified taxa within the two taxonomic levels mentioned above. The RDP categorizer function gives lists of counts for nearly 60 phyla and over 350 families (in addition to class, order, and genus level information). In addition to counts that are considered identified to a known taxon (i.e., a definite match to a taxon in the database), the categorizer also gives the number of unclassified sequences in a sample at a specific level. To compare the Newton et al. [38] summary of lake bacterioplankton to the present study, we converted the phylum level data in their Figure 2 into percent values for the short reads dataset in that figure. We also converted the overall quantities of phylum level data in our study into percentages of overall identifications. These lists of phyla and the percentage of time they occur in the Newton et al. [38] dataset and our meta-analysis were then graphed and the results appear in Fig. 2.

Taxon richness was reviewed at the phylum and family levels across geographic regions (both at the global and Brazil drainage levels) and between lotic and lentic systems using R [46]. These differences were visualized using box-and-whisker plots and tested for significance with Kruskal-Wallis tests, as data were largely nonparametric. Pairwise comparisons were then conducted using the PMCMR package’s function “posthoc.kruskal.nemenyi.test.” Statistical significance was set at *P* ≤ 0.05.

We used the counts of classified versus unclassified sequences to obtain a ratio of unidentified to identified taxa in all samples. Each unidentified taxon should generally be phylogenetically sister to (i.e., divergent from) an identified taxon (Supplemental Fig. 2A). As is standard taxonomic practice, samples like this require either expanding the definition of the identified taxon or describing a new taxon. Either scenario requires taxonomic expansion; we accordingly feel this is a meaningful addition in terms of novel biodiversity. It is worth noting that given the phenetic-based methods utilized in the RDP, it is occasionally possible for a named unclassified taxon to be an individual with much molecular change (Supplemental Fig. 2B), which similarly requires redefinition or taxonomic splitting of the original taxon. This ratio was then used to compare and quantify the degree of unclassified taxa at each of the global sites. Unclassified taxa at the phylum level refer to classes and unclassified taxa at the family level refer to genera. Heatmaps were used to visualize trends in appearance of novel taxonomic units for all of the sites in the study compared to each other and for the Brazilian subset of sites compared to each other.

### Nonmetric Multidimensional Scaling

NMDS ordinations were produced at the phylum and family levels using the “metaMDS” function in the vegan package [40] in R [46], with “trymax” set at 1000 (rerun if necessary to reach convergence). Analyses were not conducted at the genus or species level due to lack of named resolution in the RDP classifier. Dissimilarity matrices used for the NMDS analyses were produced using both standard taxon by site data and generalized UniFrac distances via the GUniFrac package and the eponymous function [12]. Details about UniFrac and NMDS analysis are as follows. Generalized UniFrac was used as it has increased power to detect changes across a larger swath of abundances than traditional or weighted UniFrac dissimilarities [12]. To create generalized UniFrac dissimilarity matrices, a backbone phylogeny was produced in PAUP* [54] for both the phylum level and the family level by downloading 16S sequences for all of the 56 phyla that are identified in the RDB classifier and all of the 363 families that are identified in the RDB classifier. The phylogenies for both taxonomic levels were then made ultrametric for analyses using the “chronos” function in the APE package [43]. Standard data were analyzed with all data as well as with rare taxa (found at <5 % of sites) removed, as is common for this type of analysis [33]. Standard error ellipses were displayed using the function “ordiellipse.” Environmental variables and measures of diversity were tested for correlations with the NMDS ordinations using the “envfit” function with 1000 permutations: the global generalized UniFrac ordination was tested for taxon richness only, while the Brazil generalized UniFrac dataset tested 22 environmental variables. Significantly correlated vectors for the Brazil dataset were then visualized on the NMDS ordinations.

Additionally, multivariate tests were conducted using PERMANOVA analyses with the “adonis” function with 1000 permutations. Analyses focused on community composition differences between global geographic locations, lotic versus lentic, and floodplain sites in Brazil (i.e., those variables focused on for visualization in ordination). The assumption of multivariate homogeneity of group dispersions was tested using the “betadisper” function.

## Results

### Analysis of Global Freshwater Bacterioplankton Diversity Compared to Brazil

We first demonstrate that our meta-analysis dataset and the Newton et al. [38] meta-analysis have similar patterns of diversity at the phylum level. The Newton et al. [38] review focused on amplified and cloned datasets for worldwide lake systems, while our study focused on short read amplicon data (mostly 454 datasets). The results of the comparison are shown in Fig. 2. As in the Newton et al. [38] study, the major phyla in our lake samples were Proteobacteria, Actinobacteria, Bacteriodetes, Verrucomicrobia, and Cyanobacteria. In general, when a phylum was not shown to exist or existed in very low quantity in the Newton et al. [38] study, we also observed low or lack of existence in our meta-analysis. One slight difference between our study and that of Newton et al. [38] is that they reported about 5 % of their sequences as unidentifiable at the phylum level, whereas we report no unidentified phyla. This difference is more than likely caused by the increase in precision of the databases used to identify sequences in environmental DNA studies from 2011 to 2016.

We next characterized the bacterial diversity of the Brazilian floodplain lakes in a global context. Supplemental Table 1 shows the distribution of sites and the number of samples obtained from Brazilian sites for the global comparisons we accomplished. We included the 58 samples from the present study to assess the diversity and community composition of the Brazilian samples in comparison to samples from other geographic regions (Supplemental Tables 1 and 2). We show results for sequences that could be assigned to the specified phylum and family levels for comparisons. We point out two limitations of the meta-analysis. First, for the southern Africa geographic region, the sample sizes are small compared to the other areas reviewed. In addition, at these southern Africa sites, there is a mixture of water and sediment in the samples. We also point out that while we are comparing global environmental samples, we do not intend for this analysis to be a definitive description of global diversity. Rather, characterization of this global set of environmental samples was accomplished to establish a context for the bacterioplankton diversity of Brazilian freshwater systems.

**Fig. 3:**
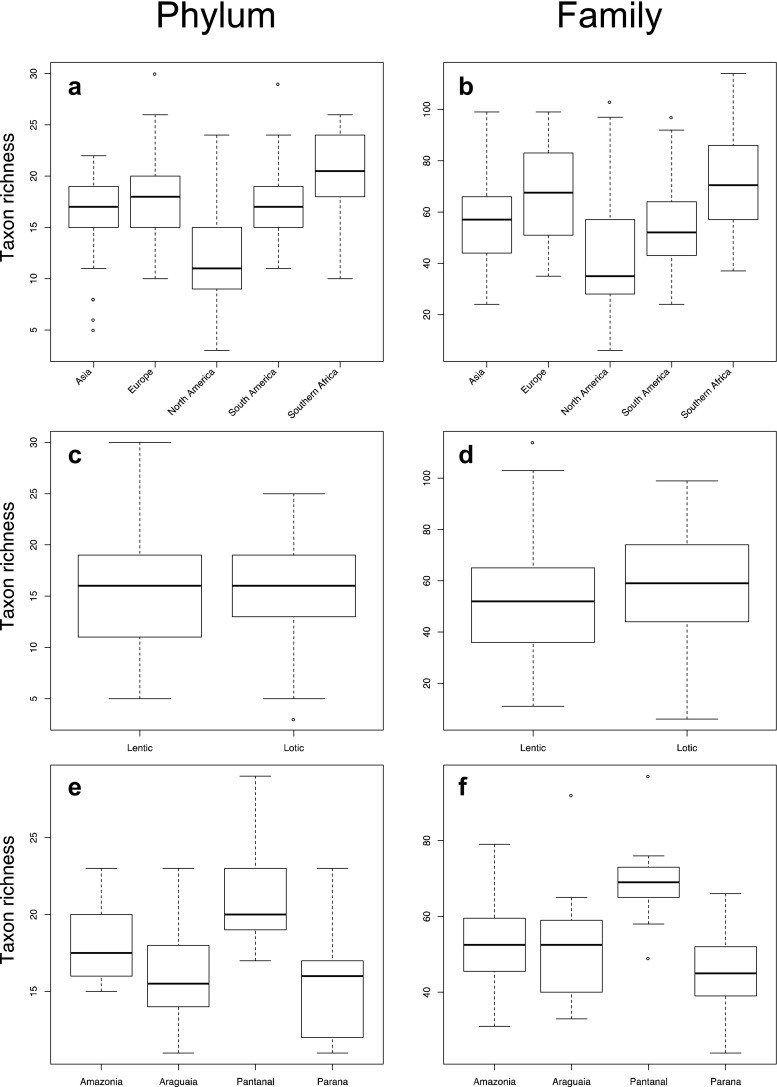
*Box-and-whisker plots* of taxon richness at the phylum and family levels for global comparisons of regions (**a**, **b**), global comparisons of lotic versus lentic systems (**c**, **d**), and comparisons of Brazilian floodplain lake sites by drainage system (**e**, **f**). The *box* represents the middle quartiles separated by the median, *whiskers* represent values up to 1.5× the interquartile range, and *dots* represent outliers

Taxon richness at both the phylum and family levels was found to be disproportionate between geographical regions (Fig. 3a, b, Supplemental Table 3, *P* < 0.001). North America was significantly lower in taxon richness than all sites. At the family level, South America has significantly lower taxon richness than Europe. In comparisons of lotic and lentic sites in the global dataset, taxon richness was similar at the phylum (*P* = 0.603) but significantly higher for lotic sites at the family (*P* = 0.009) levels (Fig. 3c, d). Finally, among the sites in Brazil, significant differences (*P* < 0.001) at both the phylum and family levels (Fig. 3e, f) were detected. Specifically, the Pantanal samples differed significantly at both taxonomic ranks (phylum and family) from all other Brazilian sites (*P* < 0.05), with the exception of the Amazon at the phylum level.

Our analysis suggests that global floodplain systems have 12 phyla that form the components of the bacterioplankton assemblage in such systems, with Proteobacteria being abundant across all sites on the globe, albeit at slightly lower frequencies for Brazil (South America; Fig. 2, Supplemental Fig. 3). Other phyla, like Cyanobacteria, Bacteroidetes, Actinobacteria, Proteobacteria, and Verrucomicrobia, were found to be major components of freshwater systems at most localities.

South America stands out globally with respect to two features of phylum level diversity. First, the Brazilian lake sites appear to have a higher proportion of Cyanobacteria and fewer Proteobacteria than other global locations. Second, in South America, Actinobacteria were more plentiful than the Bacteroidetes. Whereas most sites in Asia and southern Africa appear to have more Bacteroidetes relative to Actinobacteria, the European and North American sites appear to have relatively equal amounts of these two phyla.

Abundances of families indicate some striking differences in taxonomic makeup for Brazil relative to the rest of the global locations (Supplemental Fig. 4). First, while most global locations had Flavobacteriaceae as a major component of the freshwater systems, Brazilian lakes had low numbers of Flavobacteriaceae. Secondly, unlike most other river systems, one of the major components of the sampled lakes from South America was “Family II” in the phylum Cyanobacteria. Third, compared with other localities across the world, Brazilian freshwater systems had a greater abundance in a larger number of families in the Bacteria and Archaea, which included Opitutaceae, Burkholderiaceae, Acetobacteraceae, Methylococcaceae, and “Family I” (in the phylum Cyanobacteria).

**Fig. 4:**
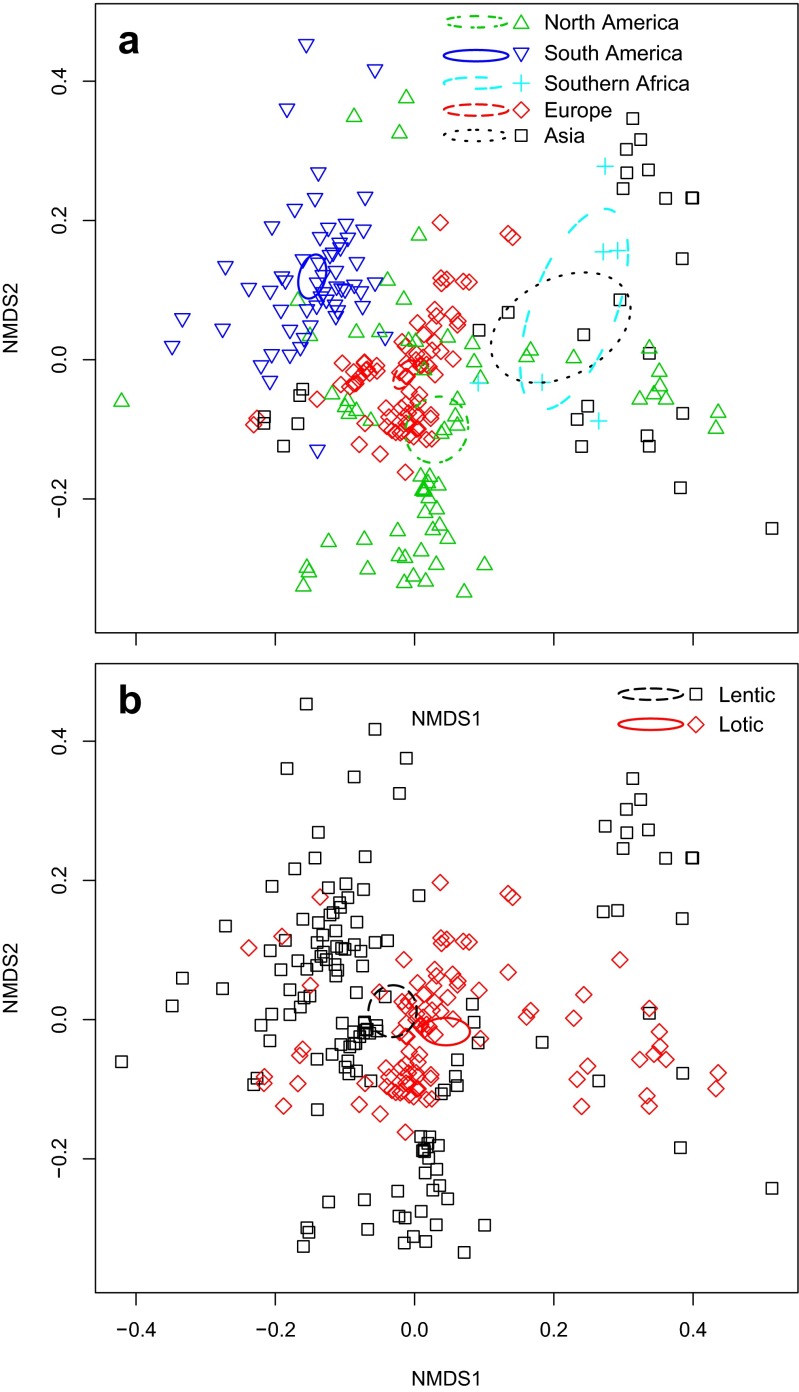
Nonmetric multidimensional scaling (NMDS) ordinations of global sites using generalized UniFrac distances of bacterioplankton identified to the family level, **a** highlighting the broad geographic area where a sample was located and **b** highlighting whether the sample was from a lotic and lentic system. *Ellipses* are for standard errors

We explored the community composition and ecological drivers of the distributions using NMDS, with several dissimilarity matrices. For this paper, we focus on the family level data analyses using a generalized UniFrac dissimilarity matrix (stress = 18.8). Figure 4 shows the NMDS ordination for the global dataset (for results at the phylum level, see Supplemental Fig. 5). Alternative analyses (i.e., standard dissimilarities at the phylum and family levels, with all data and with rare taxa removed) are included in Supplemental Fig. 6. It is clear from these analyses that Brazil occupies a distinct, largely nonoverlapping portion of ordination space. In contrast, North America appears to cover a much broader swath of ordination space, as do the six samples from South Africa. Other geographic areas were more moderate with respect to the degree of divergence.

Figure 4b focuses on visualizing which sites were lotic and lentic. Although overlapping, these two general ecotypes do roughly inhabit different halves of the ordination space. PERMANOVA analyses (comparisons of global sites and lotic vs. lentic) were all significant but with moderate to low fit (Supplemental Table 4). We point out that only the Brazil analyses met the PERMANOVA’s assumption of multivariate homogeneity of group dispersions. Geographic location had by far the strongest fit for the global dataset, with Brazil being substantially different from all other locations. Taxon richness is significantly (*P* < 0.001) correlated, but weakly fitted with our global dataset’s community composition (Supplemental Table 5).

### Analysis of Brazilian Lake Bacterioplankton Diversity

The analysis of the sites within Brazil was accomplished with the same data as in the global study. Because we had more complete metadata for these sites, we were able to examine the correspondence of several ecological factors to community composition across the 58 collection sites. Lake conditions among the four floodplain systems were slightly acidic (avg. pH 6.7), aerobic (avg. % DO 76.5 %; range 17–156 %), turbid (Secchi 0.4–0.8 m), average in chlorophyll measurement (21.9 mg/L), low in dissolved phosphorus (12–17 μg/L), and warm (avg. temperature 28.2 °C). Notable freshwater lake system diversity existed in the area of the Amazon River sampled by this expedition in that chlorophyll-*a* readings were twice as high as the average reading (48.7 mg/L), the implications of which reverberate through the doubly high TN (2579 vs. avg 1100 μg/L) and TP (114 vs. 55–83 μg/L average). The highest ammonia readings were found in the Pantanal area sampled (55 μg/L).

The heatmaps for the Brazilian sites show that in general at the level of phyla (Supplemental Fig. 7), the sites are fairly similar. One notable exception is at the Pantanal sites where Verrucomicrobia are a very minor component, whereas the other three Brazilian localities have significant numbers of Verrucomicrobia. Specifically, while there are sites from the other three freshwater systems that lack Verrucomicrobia, all Pantanal sites lack representatives from this phylum in any substantive amount. It also appears that Amazon sites are the only ones of the four floodplain lake systems to have substantive amounts of Acidobacteria as part of their bacterial assemblages. Finally, at the Pantanal sites, there was a larger proportion of Proteobacteria than was found at the other sites. At the family level, the heatmap results (Supplemental Fig. 8) suggest that Pantanal sites have fewer “Family II” Cyanobacteria in comparison to the other three floodplain lakes.

**Fig. 5:**
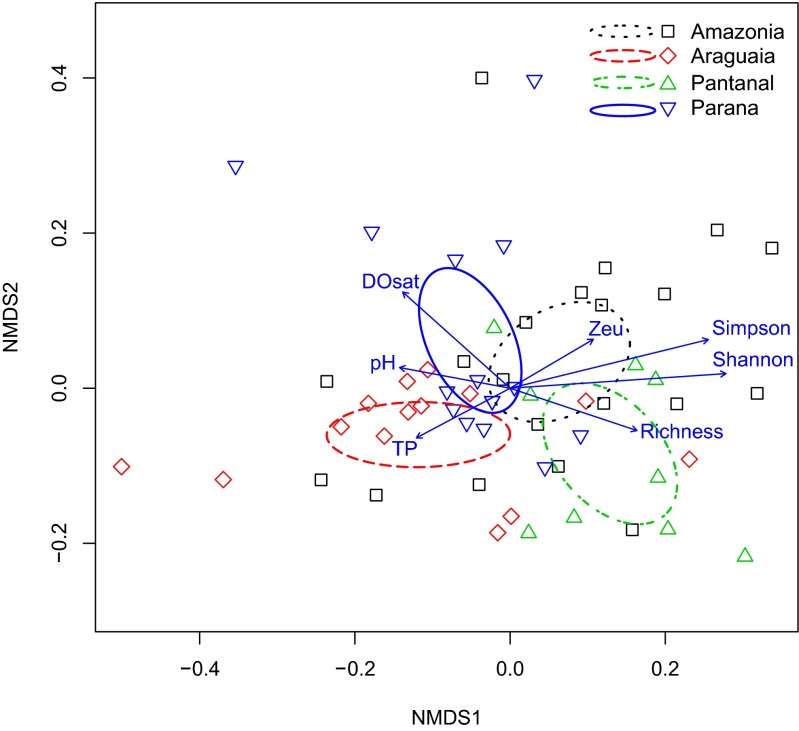
Nonmetric multidimensional scaling (NMDS) ordinations of Brazilian floodplain lake sites using generalized UniFrac distances of bacterioplankton identified to the family level, highlighting the drainage system where a sample was located. *Ellipses* are for standard errors. *Vectors* represent environmental variables that significantly correlated with the ordination space, with length corresponding to the strength of the correlation

The NMDS ordination of the Brazilian floodplain lakes with generalized UniFrac dissimilarities at the family level (stress = 18.8) is shown in Fig. 5. Results for other Brazilian NMDS ordinations are shown in Supplemental Fig. 9. Although the different drainages overlap, they do generally occupy their own portion of ordination space. Supplemental Table 5 reviews the environmental variables we examined for correlations with the generalized UniFrac ordination; significant variables are visualized in Fig. 5. It appears that saturated dissolved oxygen, taxon richness, Shannon diversity, Simpson diversity, pH, total phosphate (TP), and euphotic depth (Zeu) significantly correlate to the ordination space. The Pantanal appears to be positively correlated with increased taxon richness. The Araguaia seems to be positively correlated with TP. The Paraná looks to be positively correlated with measures of DO. The Amazon appears to positively correlate with euphotic depth. Both diversity measures and pH appear to correlate with differences along the NMDS1 axis.

### Analysis of Unclassified Taxa Among the Global Freshwater Bacterioplankton Diversity

**Fig. 6:**
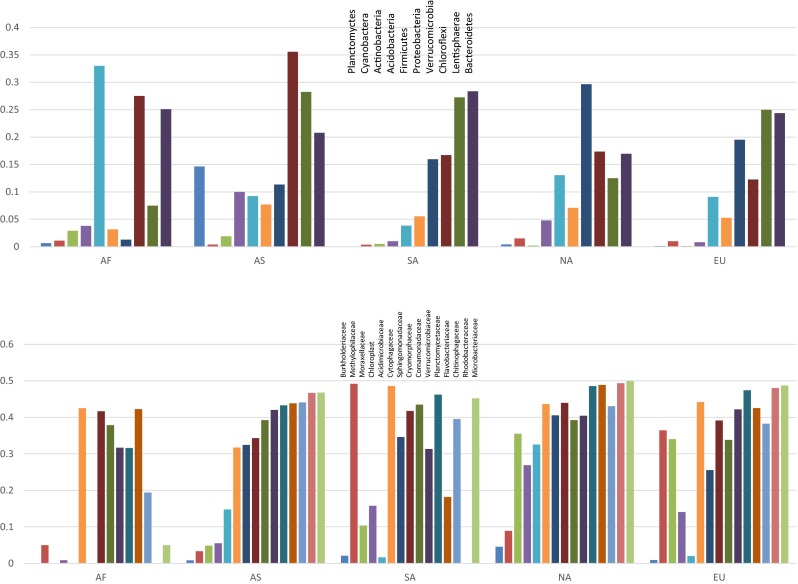
*Bar graphs* showing the percentage of taxon counts that were unidentified at the phylum (*top*) and family (*bottom*) level for the indicated taxa. For information on other phyla and families, consult Supplemental Tables 6, 7A, and 7B. The continental abbreviations are as follows: *NA* North America, *SA* South America, *EU* Europe, *AS* Asia, *AF* Africa

In the following analysis of the distribution of unidentified taxa at globally distributed sites, we have examined the amplitude of unidentified taxa below two levels—phylum and family. Brazilian sites have less unidentified taxa at both the family levels than sequences from all other sites examined (Fig. 6). This is potentially due to the fact that we sampled a very particular type of habitat (i.e., river floodplain lakes). Unidentified taxa from these sites range from zero to a few percent (several phyla at several continental locations) of the sequences being unidentified to 35 % (Chloroflexi in Asia) of the sequences being unidentified for below the phylum level and from zero to a few percent (for several families from several continental locations) to nearly 50 % (for several families from several continental locations) for below the family level (see Fig. 6).

There are 56 phyla in the RDP classifier as of 2015. We detected 12 of these phyla in substantial amounts in the global samples we examined, of which five (Verrucomicrobia, Proteobacteria, Actinobacteria, Cyanobacteria, and Bacteriodetes) comprise the grand majority of taxonomic representation. Figure 6 shows the proportion of sequences that are unidentified in the indicated phylum from the continental regions we examined for these most abundant phyla. These results indicate that novel taxa below the phylum level in the Proteobacteria, Cyanobacteria, and Actinobacteria are at or below 10 %. However, novel taxa in the Bacteroidetes and Verrucomicrobia have around 20 % unidentified taxa below the phylum level for most of the global sites (Africa is an exception). Supplemental Table 6 lists the phyla that are absent or found in extremely low numbers in the sites we examined.

At the family level for the global study, as expected, many of the sequences can be identified to genus, but a large proportion in many families cannot be identified to genus. We have examined the percentage of unidentified sequences below the family level for the 14 (top 5 % with respect to abundance) most abundant families in the samples (Burkholderiaceae, Methylophilaceae, Moraxellaceae, Acidimicrobiaceae, Cytophagaceae, Sphingomonadaceae, Cryomorphaceae, Comamonadaceae, Verrucomicrobiaceae, Planctomycetaceae, Flavobacteriaceae, Chitinophagaceae, Rhodobacteraceae, and Microbacteriaceae). These results indicate that the Brazilian samples we examined show for the most part similar levels of unidentified taxa below the family level (Fig. 6), indicating that these sites might not harbor an unusual amount of unidentified diversity below the family level.

### Analysis of Unclassified Taxa Among Brazilian Freshwater Bacterioplankton Diversity

**Fig. 7:**
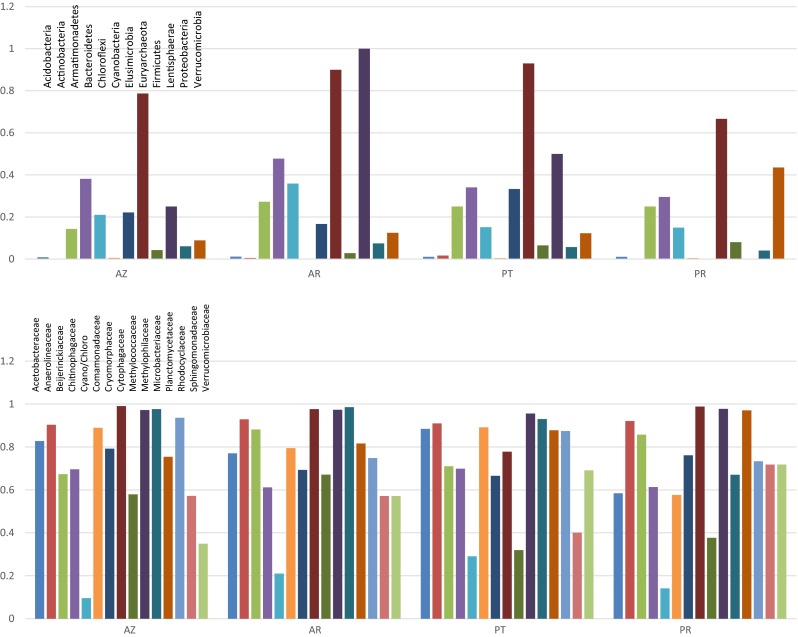
*Bar graphs* showing the percentage of taxon counts that were unidentified for Brazilian samples below the phylum level (*top*) for the indicated phyla and below family level (*bottom*) for the indicated families. For information on other phyla and families, consult Supplemental Tables 8, 9, and 10. For each plot, the abbreviations of geographical locations are as follows: *AZ* Amazon, *AR* Araguaia, *PN* Pantanal, *PR* Parana

We also compared the percentage of unclassified sequences across the Brazilian samples to determine if any floodplain lakes from a single system harbored unusual numbers of novel taxa. Figure 7 shows these comparisons at the phylum level for the four Brazilian floodplain lake systems in our study. With one exception (the Eurarchaeota), unclassified sequences for phyla are either absent or are approximately 30 % or less of the overall sequences. Five phyla, Actinobacteria, Cyanobacteria, Acidobacteria, Proteobacteria, and Firmicutes, have fewer than 5 % unclassified classes within them. All phyla that were either absent or where all sequences were classified are listed in Supplemental Table 8.

At the family level, it appears that all four floodplain lake systems have many unclassified genera in the families present at the sites. Figure 7 shows the results of this analysis for the most abundant families in the sample. In fact, most families have around 50 % of the genera unclassified within these families. Exceptions are Caulobacteraceae, Burkholderiaceae, Bacteriovoracaceae, Geobacteraceae, Acidimicrobiaceae, Mycobacteriaceae, Fusobacteriaceae, Cyanobacteria, Holophagaceae, Campylobacteraceae, Moraxellaceae, Pseudomonadaceae, Sinobacteraceae, Flavobacteriaceae, and Cyclobacteriaceae. These families in most cases have fewer than 10 % unclassified genera. Burkholderiaceae, Cyanobacteria “Family II,” Fusobacteriaceae, Acidimicrobiaceae, and Cyclobacteriaceae are notable as they have fewer than 2 % unclassified genera in these families. Information on families that had no sequences or where all sequences were classified is given in Supplemental Tables 10A and 10B, respectively.

## Discussion

The description of Bacteria primarily from floodplain lakes in four of the major river-floodplain systems in Brazil is novel and comprehensive. The patterns found at two levels of taxonomic hierarchy (phylum and family) build a picture of microbial distribution in these important, often threatened freshwater systems. As a result of our field expeditions and bioinformatics data mining, we were able to determine the bacterioplankton composition in Brazilian floodplain lake systems compared to other globally distributed freshwater systems and to expand knowledge on the biodiversity and distribution of bacterioplankton in specific floodplain ecosystems in Brazil from the Pantanal (Paraguai), Paraná, Amazon, and Araguaia Rivers.

### Comparing Brazilian Assemblages to Other Global Freshwater Bacterioplankton

We first addressed the patterns of diversity in the Brazilian sites relative to the other global sites by taking advantage of the fact that the classifiers in use will assign sequences to taxa up to a certain level where the significance of the assignment drops off. So for any given taxonomic level, there are unassigned taxa, presumably novel to our understanding of microbial diversity. Consequently, we used these unassigned sequences to assess the degree of novelty in Brazil relative to the various global sites we examined at both the phylum and family levels. We compared the number of assigned and unassigned sequences below the two levels for all of the geographic regions in our dataset.

We found a dozen prokaryotic phyla that are common in most freshwater systems, exceeding the number of phyla formerly believed to be widespread. Specifically, five phyla (Actinobacteria, Bacteroidetes, Cyanobacteria, Proteobacteria, and Verrucomicrobia) have been recognized by others to be common in freshwater communities [38]. Members of these phyla are thought to be globally distributed [3, 15, 16, 20, 26, 34, 60, 61]. Furthermore, these phyla, excluding the Verrucomicrobia, represent 95 % of the cultivated species of Bacteria [28]. A high level of diversity exists in the Gram-positive Actinobacteria and Firmicutes, the photosynthetic Cyanobacteria, and the Gram-negative Proteobacteria with different species found in soil and freshwater [23]. Though also found in soil and water, the Verrucomicrobia have few classes designated to this phylum. They are described as being ubiquitous to soil although relatively few in number [6] and common in aquatic habitats [38]. This observation is especially accurate for eutrophic bacteria recovered from heavily polluted and sulfide-rich waters [51].

Other phyla common to freshwater systems in our study were Chloroflexi, Acidobacteria, and the Lentisphaerae. Members of the monodermic Chloroflexi phylum fall into one of six weakly linked classes and the phylum was noted for having filamentous anoxygenic phototrophic members, and more recently, a good number of nonphototrophic organisms have been assigned to this group [24]. The Acidobacteria are found commonly, likely due to their physiological diversity, yet they are generally associated with soil and few exist in culture. Their existence in freshwater is becoming better described, including the Amazon River [23]. Although the Lentisphaerae are closely related to the aforementioned Verrucomicrobia as well as the Chlamydiae [13], only two orders have been described. The first is a representative from mammal and bird guts and the other is associated with marine corals, fish, and sediment. The description of these phyla is not helpful, at this point, in explaining their aquatic microbial ecology in this study.

### Brazilian Freshwater Bacterioplankton

Taxonomic richness varied substantially across global sites. Brazilian sites had similar richness compared to other global sites. The Brazilian sites are roughly from one major kind of habitat, which may explain why this region was not higher in richness. In a global scale, species diversity presents a general tendency to increase from the poles toward the equator [47]. This pattern is one of the oldest recognized in ecology, and although it is widely accepted, there is still no consensus on the processes underlying this trend [4]. However, some groups of organisms have shown variations in this pattern with higher diversity in regions far from equator (see [18, 50]). In our work, we do not see a clear increase in species richness for our more equatorial samples, but this could relate to each region having only limited sites from certain habitats sampled. In Brazil, the Pantanal was by far the richest area surveyed. This likely relates to the Pantanal’s massive size—the Earth’s largest wetland—and exceptional productivity.

For bacterioplankton composition, Brazil does appear to be globally unique, as do several other areas. This is in contrast to areas such as North America, which spread across much of ordination space. Brazil’s uniqueness likely stems from our sites being regulated by flood pulses, which contrasts sharply from other studies. Additionally, North America’s large spread in ordination space may relate to the large variety of sites scanned across several studies. Still, there were generally clearly geographically defined patterns in ordination space across all areas. Lotic and lentic sites were significant in their differences in ordination space, but very weakly so, lending support to geography being the primary predictor of site differentiation. Without further ecological data on these sites, it remains difficult to tease apart why geography plays such a critical role (e.g., is there a climactic underpinning?).

In a metacommunity perspective, our findings are highly relevant. It has been shown that the structure of aquatic communities is strongly influenced by the dispersion capacity of organisms [8], and in freshwater ecosystems, this capacity is generally inversely related to body size [8, 42]. In general, the processes related to the spatial effects seem to be more important for organisms with low dispersion capacity, while good dispersers such as microorganisms appear to be mainly controlled by local environmental conditions [8, 42]. Thus, the remarkable biogeographical patterns observed to the bacterioplankton in the present study seem to contrast the assumptions that support the metacommunities theory.

The four Brazilian river-floodplain systems were significantly but mildly differentiated. Several variables appeared to correlate with differences across ordination space, but dissolved oxygen levels, pH, and phosphate levels stood out as the strongest correlates. Dissolved oxygen has been explored for some bacteria and can influence which lineages are present (e.g., [44]). It is well known that pH is an important variable relating to the distributions of freshwater organisms such as macrophytes (e.g., [55]), macroinvertebrates (e.g., [14]), and bacteria [32, 39]. Levels of phosphate are also known to relate to bacterial composition (e.g., [7]).

### Unique Aspects of Brazilian Freshwater Lake Systems

Brazilian sites have more identified taxa below both the family and phylum levels with only about 5 % of all sequences that could not be identified below phylum and about 22 % that could not be identified below the family level. When compared to freshwater assemblages from other continents, the uniqueness of the Brazilian sites was primarily due to a higher proportion of Cyanobacteria occurring in Brazilian river floodplain lakes than in other global locations. Other patterns that emerged in the global comparison with respect to Brazil included (1) a biogeographical shift in the Bacteroidetes/Actinobacteria ratio in Brazil; (2) in general, fewer taxa in the phylum Proteobacteria in Brazil than other locations; and (3) Brazil showed a low number of Flavobacteriaceae. With respect to the Bacteroidetes/Actinobacteria ratio, in Africa and Asia, the ratio is greater than 1.0; in North America and Europe, the ratio approaches 1.0; and in South America, it is less than 1.0.

The ecological factors (listed above for the Brazilian sites), in addition to the low nutrient makeup and ephemeral nature of these lakes, may promote habitats for tolerant Cyanobacteria (e.g., [11, 37]), found commonly among the lakes in this study. The fact that the Verrucomicrobia are missing at the Pantanal, all Asian sites, and most North American sites could represent the lack of polluted and eutrophic conditions that are often associated with members of this phylum [51].

The datasets included in this study were also analyzed to the level of family. Families ubiquitous to all regions included Comamonadaceae (β-Proteobacteria), Cyanobacteria “Family II,” Chitinophagaceae (Bacteroidetes), and Planctomycetaceae (Plactomycetes). Families found in all regions except Africa included Sphingomonadaceae (α-Proteobacteria), Burkholderiaceae (β-Proteobacteria), and Acidimicrobiaceae (Actinobacteria). Only members from gamma-Proteobacteria were missing from the list of family representatives in phyla most often found in freshwater lakes (Actinobacteria, Bacteroidetes, Cyanobacteria, α-Proteobacteria, β-Proteobacteria, and the Verrucomicrobia) [38]. For the Bacteroidetes, unidentified genera are up to 50 % of the total, for instance the Cytophagaceae for the Brazilian samples.

Our previous study of the Paraná River [30] indicated that of the four identified α-Proteobacteria genera named, only *Caulobacter* was found at these sites. Though *Comamonas* was found at all sites, it was not the most abundant of the β-Proteobacteria in the freshwater systems. One of the most unusual findings was the absence of Firmicutes from a floodplain lake with unusually high humic acid content (Patos Lagoon). In contrast, Patos had a great diversity of Actinobacteria with *Dermatophilus* showing the greatest abundance of any genera (42 %). Patterns in the localized diversity at the Parana was not seen regionally in this study. It is difficult to infer ecological function or the certainty of an indicator taxon from these results, but they do serve to build a pattern of occurrence globally, as well as lend one of the first insights to organisms unique to South America.

## Conclusion

This study is the first large-scale South American freshwater eDNA investigation on prokaryotic plankton biodiversity. This study further establishes the efficacy of eDNA studies for broad, rapid comparisons of freshwater bacterioplankton. Our results find bacterioplankton taxon richness and composition to vary greatly at both the global and regional scales. Brazilian freshwater systems harbor some interesting patterns of diversity, such as low levels of Flavobacteriaceae. As with several broad geographic areas, Brazil was unique in terms of bacterioplankton composition. We also establish environmental correlates to taxon composition at a regional scale for Brazil, finding dissolved oxygen, pH, and phosphate levels to be of particular importance.

## Electronic Supplementary Material

Below is the link to the electronic supplementary material.

ESM 1 (PDF 1660 kb)
