# Supplementary material for: A Global eDNA Comparison of Freshwater Bacterioplankton Assemblages Focusing on Large-River Floodplain Lakes of Brazil
Source: Microb Ecol. 2016 Sep 9;73(1):61–74. doi: 10.1007/s00248-016-0834-5 (PMC5209421; doi:10.1007/s00248-016-0834-5)
Supplement: Supplementary file 1 — (PDF 1660 kb) [file 248_2016_834_MOESM1_ESM.pdf]

SUPPLEMENTAL MATERIAL

This document includes all of the supplemental figures and tables in Tessler and Brugler et al., - "**A Global eDNA Comparison of Freshwater Bacterioplankton Assemblages Focusing on Large-River Floodplain Lakes of Brazil**".

Supplemental Table 1. Lakes sampled, date of sample and river association are shown.

Locale	River	Date	Lab Code
Calado	Amazon	Oct.2011	9
Cadete	Amazon	Oct.2011	11
Poço Curuça	Amazon	Oct.2011	13
Poraquequara 2	Amazon	Oct.2011	15
Comprido	Amazon	Oct.2011	63
Piranha	Amazon	Oct.2011	64
Monte Cristo	Amazon	Oct.2011	65
Lagoa 5	Amazon	Oct.2011	66
Grande I	Amazon	Oct.2011	67
Fuxico	Amazon	Oct.2011	68
Grande II	Amazon	Oct.2011	69
Tatuí	Amazon	Oct.2011	70
Castanho	Amazon	Oct.2011	71
Poraquequara I	Amazon	Oct.2011	72
Poraquequara III	Amazon	Oct.2011	73
Amazon River	Amazon	Oct.2011	75
Negro River	Amazon	Oct.2011	76
Solomon River	Amazon	Oct.2011	77
Grande Ii	Amazon	May.2012	110
Tatuí	Amazon	May.2012	111
Crixas 4	Araguaia	Nov.2011	17
Goiaba	Araguaia	Mar.2012	130

Crixas I	Araguaia	Nov.2011	133
Crixas II	Araguaia	Nov.2011	134
Crixas III	Araguaia	Nov.2011	135
Japones II	Araguaia	Nov.2011	137
Montaria I	Araguaia	Nov.2011	138
Montaria III	Araguaia	Nov.2011	139
Piranha	Araguaia	Nov.2011	140
Varal	Araguaia	Nov.2011	141
Brito	Araguaia	Nov.2011	142
Comprido I	Araguaia	Nov.2011	143
Goiaba	Araguaia	Nov.2011	144
Luis Alves II	Araguaia	Nov.2011	146
Albuquerque	Pantanal	Mar.2012	29
Piúva	Pantanal	Mar.2012	31
Tuiuiu	Pantanal	Mar.2012	78
Caceres	Pantanal	Mar.2012	79
Miranda I	Pantanal	Mar.2012	80
Figueira	Pantanal	Mar.2012	84
Ilha Grande	Pantanal	Mar.2012	85
Rebojão	Pantanal	Mar.2012	86
Paraguai River	Pantanal	Mar.2012	87
Patos	Parana	Feb.2012	2
Guaraná	Parana	Sept.2011	3
Porcos	Parana	Sept.2011	5

Ventura	Parana	Sept.2011	47
Peroba	Parana	Sept.2011	50
B Ipoitã	Parana	Sept.2011	51
Fechada	Parana	Sept.2011	52
Onça	Parana	Sept.2011	53
Gavião	Parana	Sept.2011	54
Aurélio	Parana	Sept.2011	55
P Garças	Parana	Sept.2011	56
M Luiza	Parana	Sept.2011	57
R Pau Véio	Parana	Sept.2011	58
Fechada	Parana	Feb.2012	93
Sumida	Parana	Sep.2011	147

Supplemental Table 2. Global data sets used in the study. References for the studies can be found by searching the various accessions.

Site	Continent	Lake/river	N	Method	Accession	BioProject	SRA Study:
South Africa	Africa	L	6	454	SRX494268	PRJNA239796	SRP039885
10 Lakes	Asia	L	14	454	SRX674047	PRJNA255556	SRP044626
Pearl River	Asia	R	15	454	SRX366257	PRJNA192558	SRP019932
Lake Ladoga	Europe	L	3	454	SRX516909	PRJNA244610	SRP041172
Danube River	Europe	R	65	Illumina	SRX665528	PRJNA256993	SRP045083
Uppsala	Europe	L	16	454	SRX039399	*	SRP005457
Lake Munson	NA	L	9	454	SRX276801	PRJNA200952	SRP022185
Lake Lanier	NA	L	6	454	SRX117025	PRJNA51219	SRP003195
Columbia River	NA	R	18	454	SRX057863	*	SRP006412
Lake Muskegon	NA	L	21	454	SRX833786	PRJNA271696	SRP051811
Lake Michigan	NA	L	15	Illumina	SRX547852	PRJNA245802	SRP041536
Brazil	SA	TS	58	454	TS	TS	TS
Total			326				

* = Not Listed

Supplemental Table 3. *P*-values from pairwise Kruskal-Wallis tests comparing taxon (phylum level, bottom left; family level, top right) richness by location. Significant values are bolded.

	Asia	Europe	North America	South America	Southern Africa
Asia	-	0.175	**0.005**	0.949	0.653
Europe	0.709	-	**< 0.001**	**0.002**	0.998
North America	**< 0.001**	**< 0.001**	-	**0.007**	**0.011**
South America	0.929	0.984	**< 0.001**	-	0.367
Southern Africa	0.512	0.845	**0.001**	0.738	-

Supplemental Table 4. Results of PERMANOVA analyses at the phylum and family levels.

	Phylum		Family	
	R^2	*P*	R^2	*P*
Location	0.24	< 0.001	0.22	< 0.001
Brazil vs. other locales	0.11	< 0.001	0.11	< 0.001
Lentic vs. lotic	0.05	< 0.001	0.06	< 0.001
Brazil floodplains	0.11	0.013	0.13	< 0.001

Supplemental Table 5. Correlations between environmental metadata and the NMDS ordination as found with "envfit" vector fitting for the global and Brazil only data sets at the family level. Significant correlations are bolded.

	R^2	P
Brazil		
Diversity, Shannon	0.62	< **0.001**
Diversity, Simpson	0.55	< **0.001**
D.O. saturated	0.28	< **0.001**
Richness	0.24	< **0.001**
pH	0.17	**0.008**
Total phosphate	0.15	**0.030**
Z. euphotic.	0.12	**0.031**
Depth	0.07	0.161
Z. max.	0.07	0.161
Chlorophyll	0.08	0.163
Dissolved inorganic nitrogen (DIN)	0.06	0.196
Total nitrogen	0.06	0.226
NH_4	0.05	0.256
NO_3	0.04	0.372
PO_4	0.03	0.403
Abundance	0.03	0.409
Conductivity	0.02	0.603
Temperature (H_2O)	0.01	0.791
Turbidity	0.00	0.920

	R^2	P
Global		
Taxon richness	0.17	< **0.001**

Supplemental Table 6. Phyla that are either represented at very low frequency or not at all in aquatic environments reviewed for global comparisons.

Acetothermia	Korarchaeota
Aminicenantes	Latescibacteria
Aquificae	Lentisphaerae
Armatimonadetes	Marinimicrobia
Atribacteria	Microgenomates
Caldiserica	Nanoarchaeota
candidate division WPS-1	Nanohaloarchaeota
candidate division WPS-2	Nitrospinae
Candidatus Calescamantes	Omnitrophica
Candidatus Saccharibacteria	Parcubacteria
Chlorobi	Poribacteria
Cloacimonetes	Spirochaetes
Crenarchaeota	SR1
Deferribacteres	Synergistetes
Deinococcus-Thermus	Tenericutes
Dictyoglomi	Thaumarchaeota
Elusimicrobia	Thermodesulfobacteria
Euryarchaeota	Thermotogae
Fibrobacteres	
Fusobacteria	
Hydrogenedentes	

Supplemental Table 7A. Families where all sequences were classified to the genus level in global comparisons.

AcidimicrobineaeIS	Gallionellaceae
Acidothermaceae	Gemmatimonadaceae
Alcanivoracaceae	Granulosicoccaceae
Armatimonadaceae	Haliangiaceae
Aurantimonadaceae	Halothiobacillaceae
Bacteroidaceae	Iamiaceae
Bdellovibrionaceae	Kofleriaceae
Brucellaceae	Legionellaceae
Chthonomonadaceae	Leptospiraceae
Clostridiales_IS XII	Methylobacteriaceae
Conexibacteraceae	Moritellaceae
Corynebacteriaceae	Nakamurellaceae
Deferribacteraceae	Nitriliruptoraceae
DeferribacteralesIS	Nitrospinaceae
Desulfomicrobiaceae	Parvularculaceae
Dietziaceae	Phaselicystidaceae
Family I (Cyano)	PseudomonadalesIS
Family III (Cyano)	Psychromonadaceae
Family IV (Cyano)	Rhizobiaceae
Family IX (Cyano)	Ruaniaceae
Family V (Cyano)	Rubrobacteraceae
Family VI (Cyano)	Sanguibacteraceae
Family VIII (Cyano)	SAR11
Family X (Cyano)	Shewanellaceae
Family XI (Cyano)	Sneathiellaceae
Family XII (Cyano)	Solirubrobacteraceae
Family XIII (Cyano)	Sporichthyaceae
Fimbriimonadaceae	Streptosporangiaceae
Francisellaceae	Syntrophorhabdaceae
Gaiellaceae	Thermoleophilaceae
	Thiotrichaceae

Supplemental Table 7B. Families where no assignments were made (neither identified nor unidentified) in global comparisons.

Acanthopleuribacteraceae	Ferritrophicaceae
Acidithiobacillaceae	Ferrovaceae
Actinomycetaceae	Heliobacteriaceae
AlteromonadalesIS	Hydrogenimonaceae
Anaplasmataceae	Hydrogenothermaceae
Aquificaceae	Idiomarinaceae
AquificalesIS	IS III
Bartonellaceae	IS IV
Beutenbergiaceae	Kiloniellaceae
Bogoriellaceae	Kordiimonadaceae
Cardiobacteriaceae	Litoricolaceae
Celerinatantimonadaceae	Magnetococcaceae
Clostridiaceae 3	Natranaerobiaceae
Clostridiaceae 4	OceanospirillalesIS
Clostridiales_IS III	Oleiphilaceae
Clostridiales_IS IV	Orbaceae
Clostridiales_IS XIV	Pasteurellaceae
Clostridiales_IS XVI	Peptococcaceae 2
Clostridiales_IS XVII	Peptococcaceae I
Clostridiales_IS XVIII	Procabacteriaceae
ClostridialesIS	Promicromonosporaceae
Cohaesibacteraceae	Saccharospirillaceae
Dermabacteraceae	Salinisphaeraceae
Desulfarculaceae	Spirillaceae
Desulfohalobiaceae	Succinivibrionaceae
Desulfonatronaceae	Sutterellaceae
Desulfurellaceae	Thermithiobacillaceae
Desulfurobacteriaceae	Thermotogaceae
Ferrimonadaceae	ThermotogalesIS

Supplemental Table 8. List of phyla with no sequences (left) and phyla where all sequences were classified (right) for the four river systems in Brazil.

No sequences	All sequences classified
Aquificae	BRC1
Atribacteria	candidate division WPS-1
Caldiserica	candidate division WPS-2
Candidatus Calescamantes	candidate division ZB3
Chrysiogenetes	Candidatus Saccharibacteria
Cloacimonetes	Chlamydiae
Dictyoglomi	Chlorobi
Korarchaeota	Crenarchaeota
Marinimicrobia	Deferribacteres
Microgenomates	Deinococcus-Thermus
Nanoarchaeota	Fibrobacteres
Nanohaloarchaeota	Fusobacteria
Nitrospinae	Gemmatimonadetes
Omnitrophica	Hydrogenedentes
Poribacteria	Ignavibacteriae
Thaumarchaeota	Latescibacteria
Thermodesulfobacteria	Lentisphaerae
Thermotogae	Nitrospirae
Tenericutes	Parcubacteria
Synergistetes	Planctomycetes
	Spirochaetes
	SR1

Supplemental Table 9. Families with no sequences in Brazil river systems.

Acanthopleuribacteraceae	Clostridiales_IS XIV	Flammeovirgaceae	Nakamurellaceae	Sanguibacteraceae
Acidithiobacillaceae	Clostridiales_IS XVI	Francisellaceae	Nannocystaceae	Segniliparaceae
Acidothermaceae	Clostridiales_IS XVII	Geodermatophilaceae	Natranaerobiaceae	Shewanellaceae
Actinomycetaceae	Clostridiales_IS XVIII	Glycomycetaceae	Nautiliaceae	Sneathiellaceae
Actinopolysporaceae	ClostridialesIS	Granulosicoccaceae	Nitriliruptoraceae	Solirubrobacteraceae
Actinospicaceae	Cohaesibacteraceae	Hahellaceae	Nitrosomonadaceae	Spirillaceae
Alcanivoracaceae	Colwelliaceae	Haliangiaceae	Nitrospinaceae	SpirochaetalesIS
Alteromonadaceae	Corynebacteriaceae	Halomonadaceae	Nocardiaceae	Streptomycetaceae
AlteromonadalesIS	CorynebacterineaeIS	Halothiobacillaceae	Nocardioidaceae	Streptosporangiaceae
Anaplasmataceae	Coxiellaceae	Heliobacteriaceae	Nocardiopsaceae	StreptosporangineaeIS
Aquificaceae	Cryptosporangiaceae	Hydrogenimonaceae	Oceanospirillaceae	Succinivibrionaceae
AquificalesIS	Deferribacteraceae	Hydrogenophilaceae	OceanospirillalesIS	Sutterellaceae
Armatimonadaceae	DeferribacteralesIS	Hydrogenothermaceae	Oleiphilaceae	Syntrophobacteraceae
Aurantimonadaceae	Defluviitaleaceae	Hyphomonadaceae	Orbaceae	Syntrophomonadaceae
Bacteroidaceae	Demequinaceae	Iamiaceae	Parvularculaceae	Syntrophorhabdaceae
BacteroidalesIS	Dermabacteraceae	Idiomarinaceae	Pasteurellaceae	Thermithiobacillaceae
Bartonellaceae	Dermacoccaceae	IS III	Patulibacteraceae	Thermoleophilaceae
Beutenbergiaceae	Dermatophilaceae	IS IV	Peptococcaceae 1	Thermomonosporaceae
Bifidobacteriaceae	Desulfarculaceae	IS XI	Peptococcaceae 2	Thermotogaceae
Bogoriellaceae	Desulfobacteraceae	Jiangellaceae	Peptococcaceae I	ThermotogalesIS
Brachyspiraceae	Desulfobulbaceae	Jonesiaceae	Peptostreptococcaceae	Thiotrichaceae
Brevibacteriaceae	Desulfohalobiaceae	Kiloniellaceae	Phaselicystidaceae	ThiotrichalesIS
Brevinemataceae	Desulfomicrobiaceae	Kineosporiaceae	Phyllobacteriaceae	Tsukamurellaceae
Brucellaceae	Desulfonatronaceae	Kordiimonadaceae	Piscirickettsiaceae	Vibrionaceae
Caldisericaceae	Desulfovibrionaceae	Lachnospiraceae	Procabacteriaceae	Xanthobacteraceae
Cardiobacteriaceae	Desulfurellaceae	Legionellaceae	Promicromonosporaceae	
Catenulisporaceae	Desulfurobacteriaceae	Leptospiraceae	Propionibacteriaceae	
Celerinatantimonadaceae	Desulfuromonadaceae	Leptotrichiaceae	Pseudoalteromonadaceae	
Cellulomonadaceae	Dietziaceae	Litoricolaceae	PseudomonadalesIS	
Chromatiaceae	Ectothiorhodospiraceae	Magnetococcaceae	Pseudonocardiaceae	
Chrysiogenaceae	Erythrobacteraceae	Marinilabiaceae	Psychromonadaceae	
Chthonomonadaceae	Eubacteriaceae	Marinilabiliaceae	Rarobacteraceae	
Clostridiaceae 2	Euzebyaceae	Mariprofundaceae	Rhizobiaceae	
Clostridiaceae 3	Family V (Cyano)	Methylobacteriaceae	RhizobialesIS	
Clostridiaceae 4	Family VII (Cyano)	Micrococcaceae	Rhodothermaceae	
Clostridiales_IS III	Family VIII (Cyano)	MicrococcineaeIS	Rikenellaceae	
Clostridiales_IS IV	Family XII (Cyano)	Micromonosporaceae	Ruaniaceae	
Clostridiales_IS XI	Ferrimonadaceae	Mooreiaceae	Rubrobacteraceae	
Clostridiales_IS XII	Ferritrophicaceae	Moritellaceae	Saccharospirillaceae	
Clostridiales_IS XIII	Ferrovaceae	Myxococcaceae	Salinisphaeraceae	

Supplemental Table 10A. Families with no sequences in Brazil river systems.

Acanthopleuribacteraceae	Clostridiales_IS XIV	Flammeovirgaceae	Nakamurellaceae	Sanguibacteraceae
Acidithiobacillaceae	Clostridiales_IS XVI	Francisellaceae	Nannocystaceae	Segniliparaceae
Acidothermaceae	Clostridiales_IS XVII	Geodermatophilaceae	Natranaerobiaceae	Shewanellaceae
Actinomycetaceae	Clostridiales_IS XVIII	Glycomycetaceae	Nautiliaceae	Sneathiellaceae
Actinopolysporaceae	ClostridialesIS	Granulosicoccaceae	Nitriliruptoraceae	Solirubrobacteraceae
Actinospicaceae	Cohaesibacteraceae	Hahellaceae	Nitrosomonadaceae	Spirillaceae
Alcanivoracaceae	Colwelliaceae	Haliangiaceae	Nitrospinaceae	SpirochaetalesIS
Alteromonadaceae	Corynebacteriaceae	Halomonadaceae	Nocardiaceae	Streptomycetaceae
AlteromonadalesIS	CorynebacterineaeIS	Halothiobacillaceae	Nocardioidaceae	Streptosporangiaceae
Anaplasmataceae	Coxiellaceae	Heliobacteriaceae	Nocardiopsaceae	StreptosporangineaeIS
Aquificaceae	Cryptosporangiaceae	Hydrogenimonaceae	Oceanospirillaceae	Succinivibrionaceae
AquificalesIS	Deferribacteraceae	Hydrogenophilaceae	OceanospirillalesIS	Sutterellaceae
Armatimonadaceae	DeferribacteralesIS	Hydrogenothermaceae	Oleiphilaceae	Syntrophobacteraceae
Aurantimonadaceae	Defluviitaleaceae	Hyphomonadaceae	Orbaceae	Syntrophomonadaceae
Bacteroidaceae	Demequinaceae	Iamiaceae	Parvularculaceae	Syntrophorhabdaceae
BacteroidalesIS	Dermabacteraceae	Idiomarinaceae	Pasteurellaceae	Thermithiobacillaceae
Bartonellaceae	Dermacoccaceae	IS III	Patulibacteraceae	Thermoleophilaceae
Beutenbergiaceae	Dermatophilaceae	IS IV	Peptococcaceae 1	Thermomonosporaceae
Bifidobacteriaceae	Desulfarculaceae	IS XI	Peptococcaceae 2	Thermotogaceae
Bogoriellaceae	Desulfobacteraceae	Jiangellaceae	Peptococcaceae I	ThermotogalesIS
Brachyspiraceae	Desulfobulbaceae	Jonesiaceae	Peptostreptococcaceae	Thiotrichaceae
Brevibacteriaceae	Desulfohalobiaceae	Kiloniellaceae	Phaselicystidaceae	ThiotrichalesIS
Brevinemataceae	Desulfomicrobiaceae	Kineosporiaceae	Phyllobacteriaceae	Tsukamurellaceae
Brucellaceae	Desulfonatronaceae	Kordiimonadaceae	Piscirickettsiaceae	Vibrionaceae
Caldisericaceae	Desulfovibrionaceae	Lachnospiraceae	Procabacteriaceae	Xanthobacteraceae
Cardiobacteriaceae	Desulfurellaceae	Legionellaceae	Promicromonosporaceae	
Catenulisporaceae	Desulfurobacteriaceae	Leptospiraceae	Propionibacteriaceae	
Celerinatantimonadaceae	Desulfuromonadaceae	Leptotrichiaceae	Pseudoalteromonadaceae	
Cellulomonadaceae	Dietziaceae	Litoricolaceae	PseudomonadalesIS	
Chromatiaceae	Ectothiorhodospiraceae	Magnetococcaceae	Pseudonocardiaceae	
Chrysiogenaceae	Erythrobacteraceae	Marinilabiaceae	Psychromonadaceae	
Chthonomonadaceae	Eubacteriaceae	Marinilabiliaceae	Rarobacteraceae	
Clostridiaceae 2	Euzebyaceae	Mariprofundaceae	Rhizobiaceae	
Clostridiaceae 3	Family V	Methylobacteriaceae	RhizobialesIS	
Clostridiaceae 4	Family VII	Micrococcaceae	Rhodothermaceae	
Clostridiales_IS III	Family VIII	MicrococcineaeIS	Rikenellaceae	
Clostridiales_IS IV	Family XII	Micromonosporaceae	Ruaniaceae	
Clostridiales_IS XI	Ferrimonadaceae	Mooreiaceae	Rubrobacteraceae	
Clostridiales_IS XII	Ferritrophicaceae	Moritellaceae	Saccharospirillaceae	
Clostridiales_IS XIII	Ferrovaceae	Myxococcaceae	Salinisphaeraceae	

Supplemental Table 10B. Families in Brazil river systems with no unclassified sequences.

Acetobacteraceae	Conexibacteraceae	Gaiellaceae	Prevotellaceae
Acidimicrobiaceae	Cyclobacteriaceae	Gallionellaceae	Pseudomonadaceae
Acidimicrobineae_incertae_sedis	Cystobacteraceae	Gemmatimonadaceae	Rhodobacteraceae
Aeromonadaceae	Cytophagaceae	Geobacteraceae	Rhodobiaceae
Alcaligenaceae	Coriobacteriaceae	Helicobacteraceae	Rhodocyclaceae
Bacteriovoracaceae	Cryomorphaceae	Holophagaceae	Rhodospirillaceae
Bdellovibrionaceae	Enterobacteriaceae	Hyphomicrobiaceae	Rickettsiaceae
Beijerinckiaceae	Family I (Cyano)	Intrasporangiaceae	Ruminococcaceae
Bifidobacteriaceae	Family II (Cyano)	Kofleriaceae	Saprospiraceae
Bradyrhizobiaceae	Family III (Cyano)	Methylococcaceae	SAR11
Burkholderiaceae	Family IV (Cyano)	Methylocystaceae	Sinobacteraceae
Burkholderiales_incertae_sedis	Family IX (Cyano)	Methylophilaceae	Sphingobacteriaceae
Campylobacteraceae	Family VI (Cyano)	Microbacteriaceae	Sphingomonadaceae
Caulobacteraceae	Family X (Cyano)	Moraxellaceae	Spirochaetaceae
Chitinophagaceae	Family XI (Cyano)	Mycobacteriaceae	Sporichthyaceae
Chlorobiaceae	Family XIII (Cyano)	Neisseriaceae	Syntrophaceae
Chloroplast	Fimbriimonadaceae	Oxalobacteraceae	Xanthomonadaceae
Clostridiaceae 1	Flavobacteriaceae	Polyangiaceae	
Comamonadaceae	Fusobacteriaceae	Porphyromonadaceae	

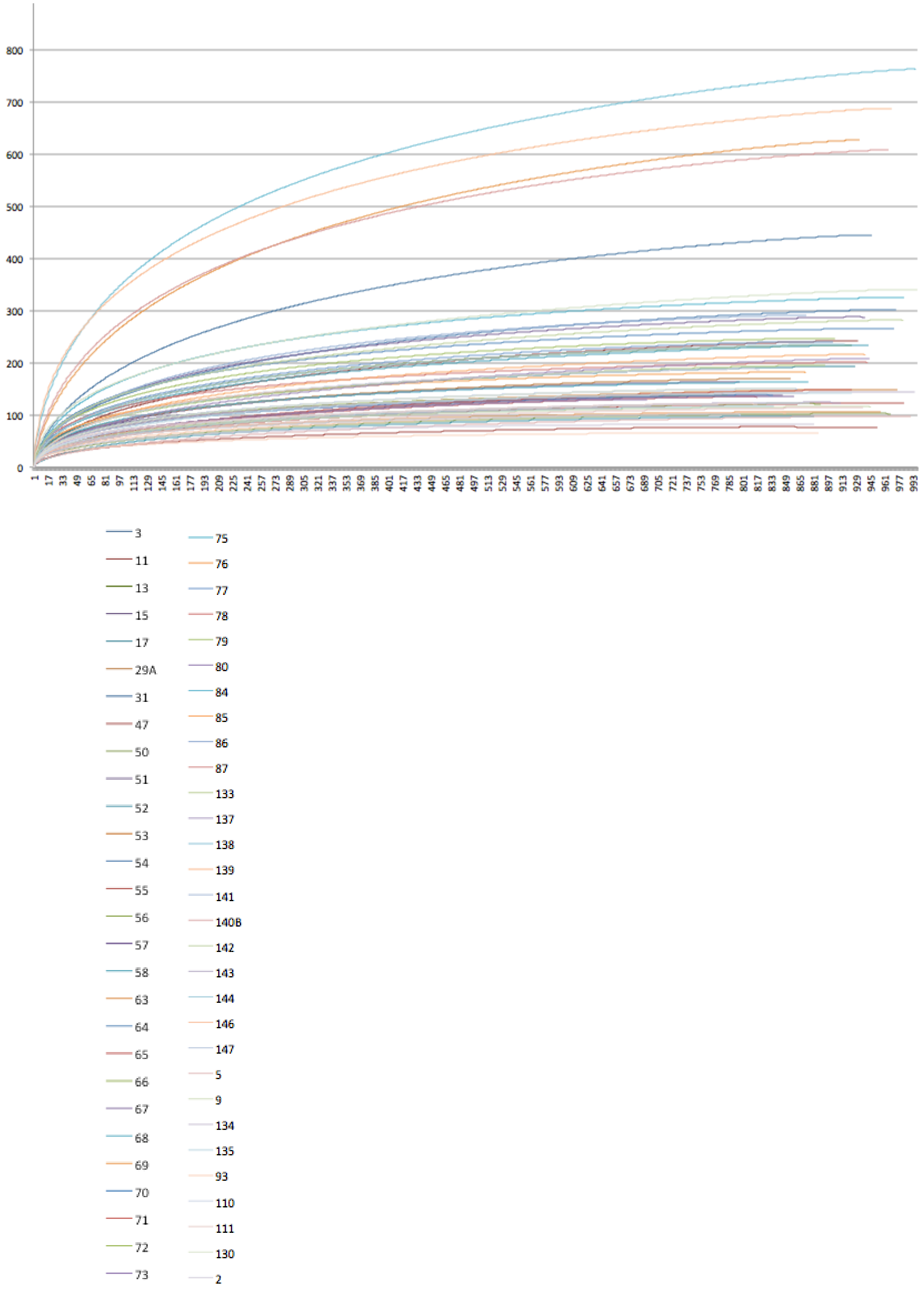

Supplemental Figure 1. Rarefaction Curves for the 58 Brazil sites at the genus level. X axis represents number of genera and Y axis represents relative reads. The line colors refer to the lab codes in Supplemental Table 1.

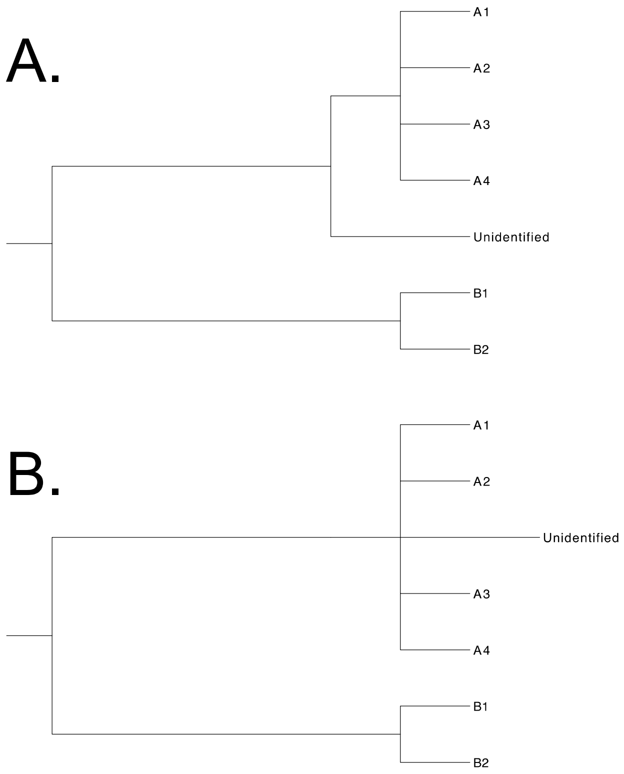

Supplemental Figure 2. Potential phylogenetic scenarios leading to unclassified taxa: A. the unidentified taxon is sister, but diverged, from named taxon “A”; B. the unidentified taxon has considerable molecular evolution from the unresolved taxon “A” individuals. Taxon “B” represents a more distant relative.

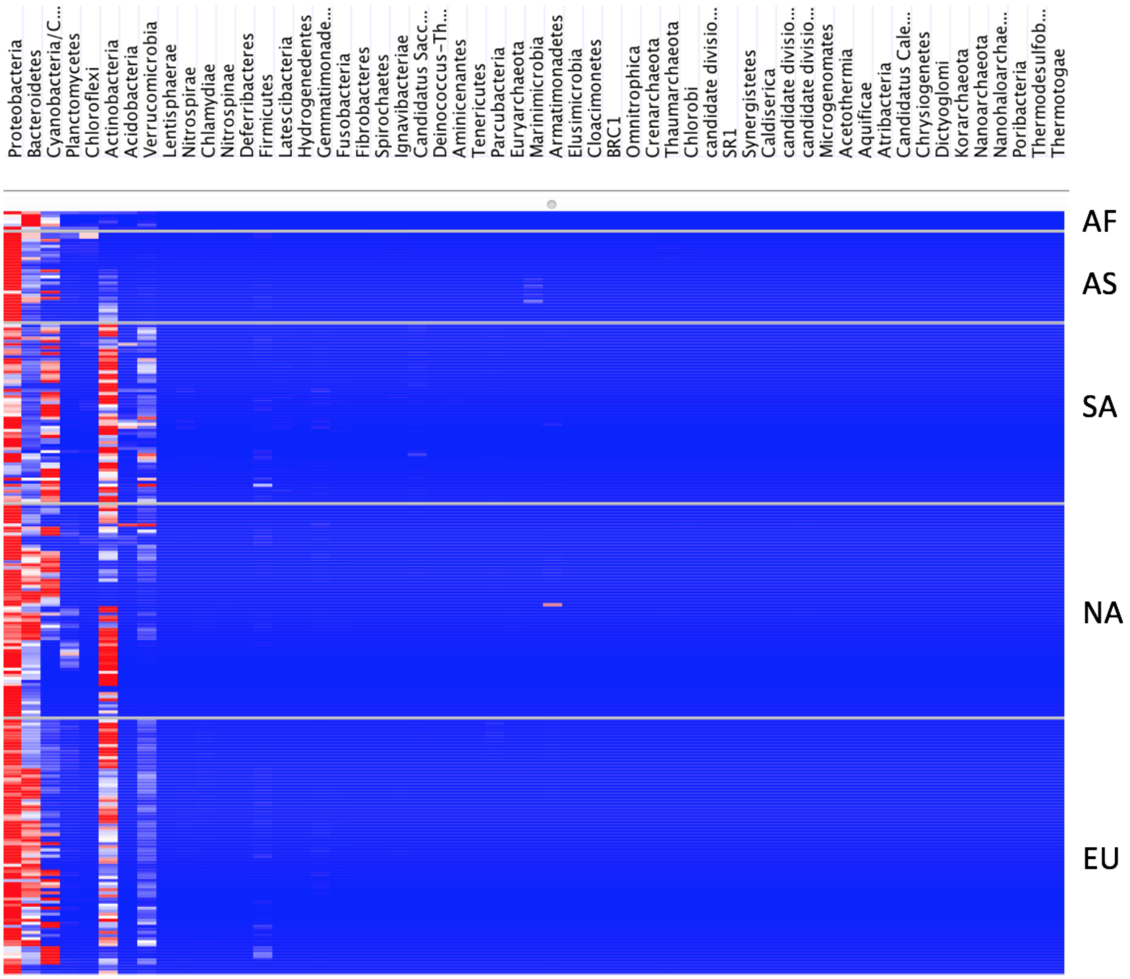

Supplemental Figure 3. Heat map at the phylum level for the global analysis. Global Geographic abbreviations are AF=Africa, AS=Asia, EU=Europe, HA=high Arctic, NA=North America and SA=South America.

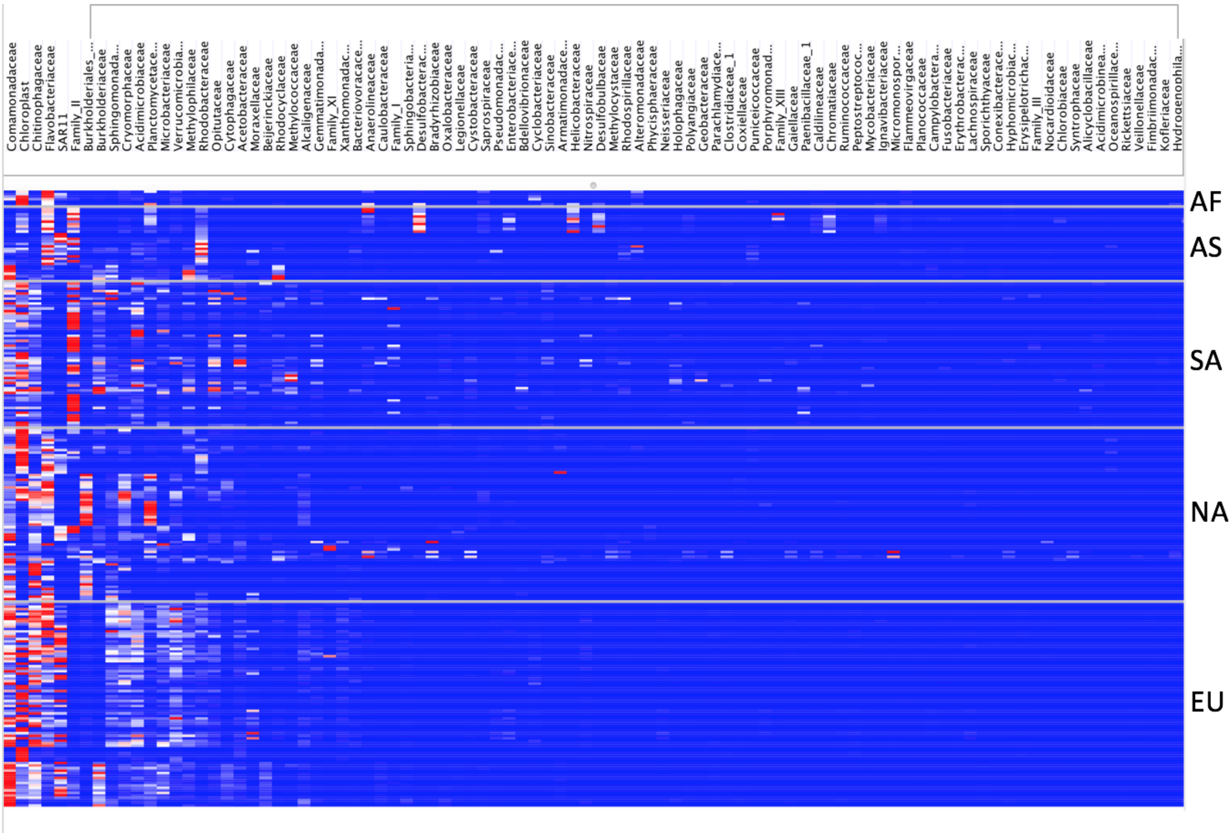

Supplemental Figure 4. Heatmap at the family level of the global analysis. Global Geographic abbreviations are AF=Africa, AS=Asia, EU=Europe, HA=high Arctic, NA=North America and SA=South America.

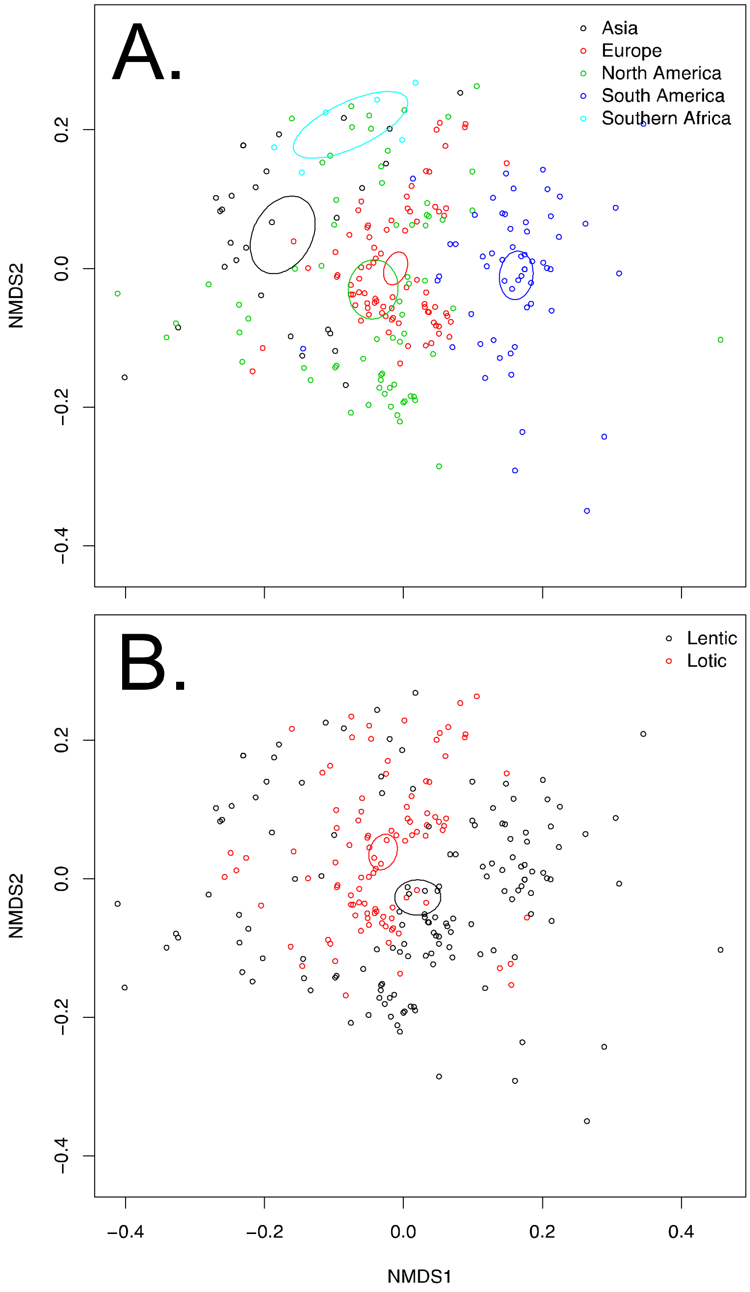

Supplemental Figure 5. Nonmetric multidimensional scaling (NMDS) ordinations of global sites using generalized UniFrac distances of bacterioplankton identified to the phylum level, with A. highlighting the broad geographic area where a sample was located and B. highlighting whether the sample was from a lotic and lentic system. Ellipses are for standard errors.

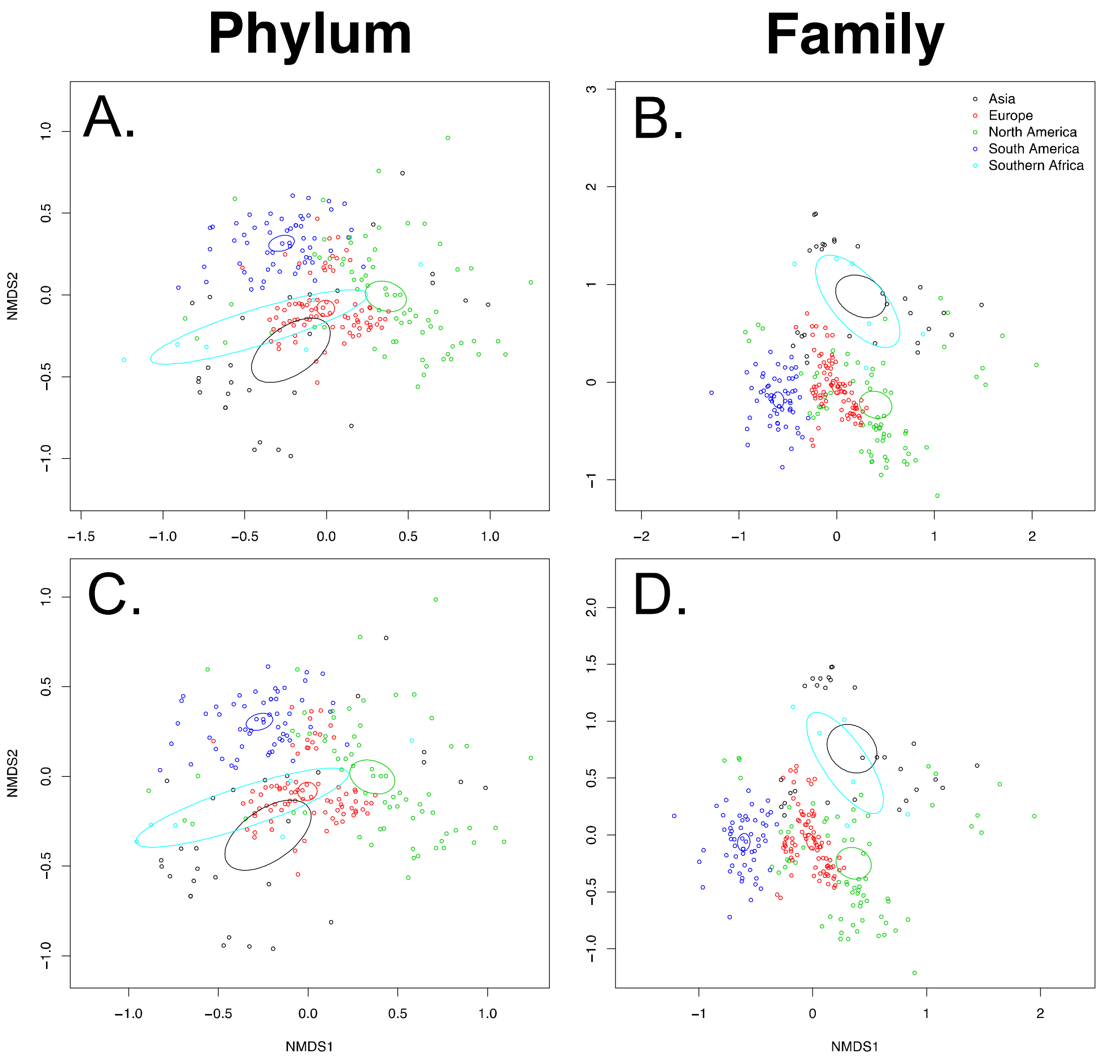

Supplemental Figure 6. NMDS analyses of standard data for the global data set at the phylum (A., C.) and family (B., D.) levels for the full data set (A., B.) and with rare species removed (C., D.). Ellipses are for standard errors.

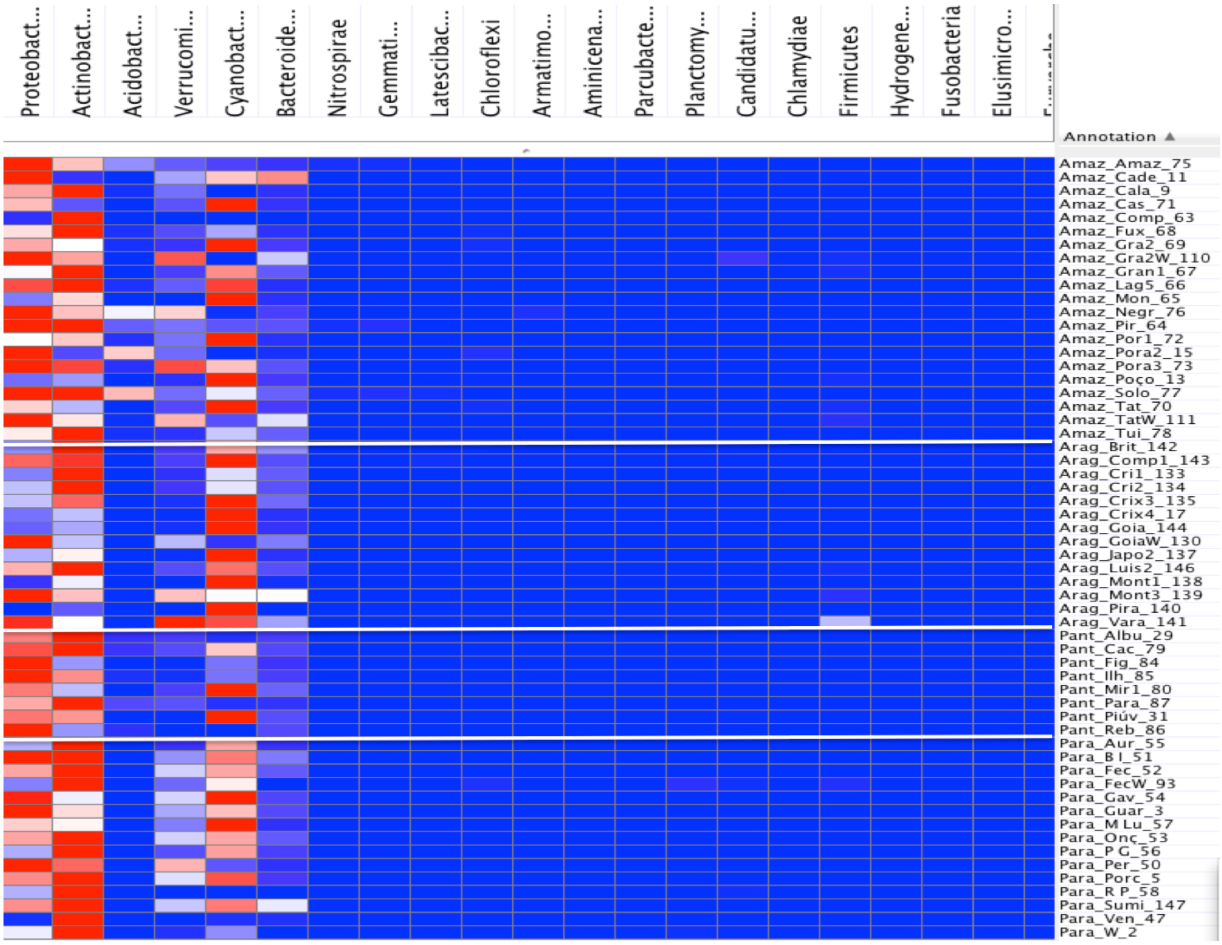

Supplemental Figure 7. Heatmap at the phylum level for the individual collecting sites for the four Brazil drainage systems. The notation on the left in the locality name indicates the major drainage system (Amaz=Amazon, Arag=Araguaia, Pant=Pantanal and Para=Paraná. The middle of the notation gives a coded specific locality in the indicated drainage system (see Supplemental Table 1) and the last part of the notation is our archival number for the locality collection.

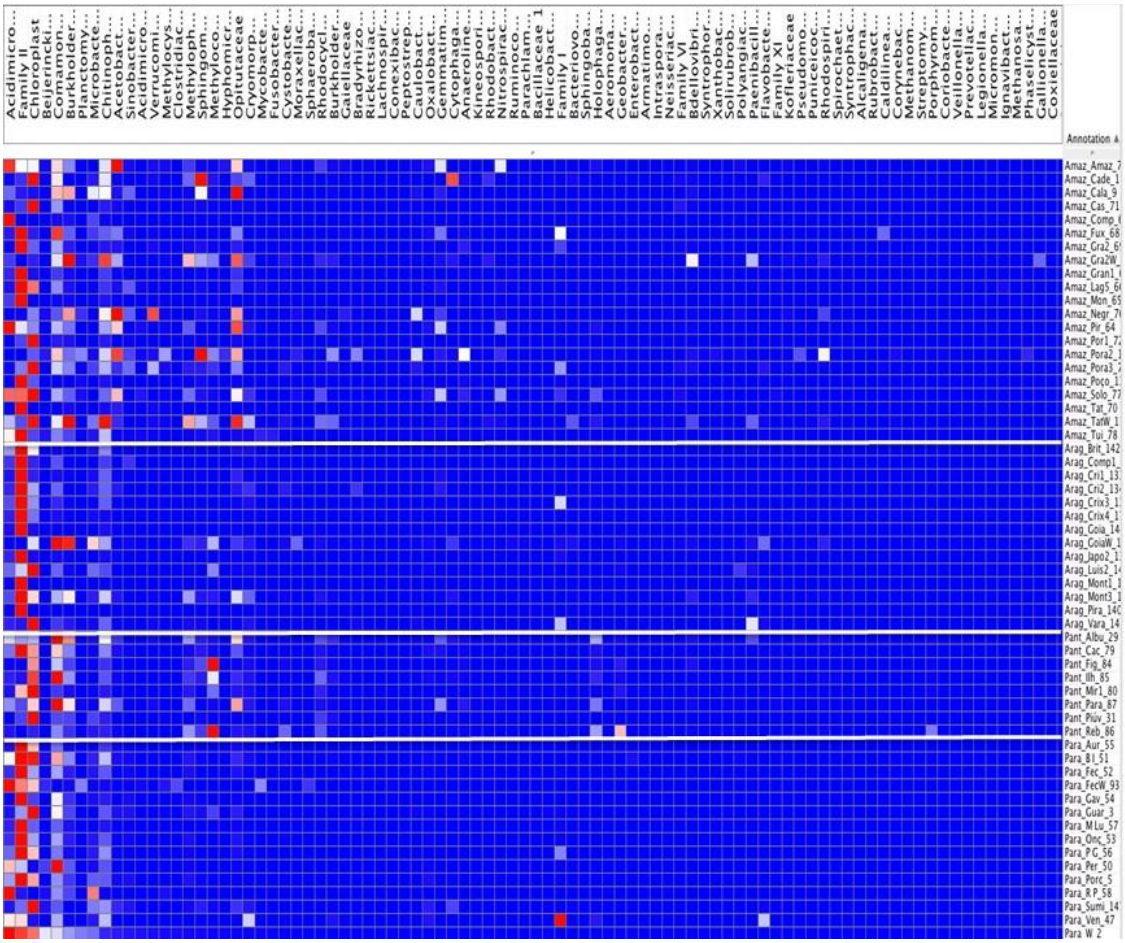

S6

Supplemental Figure 8. Heatmap at the family level for the individual collecting sites for the four Brazil drainage systems. The notation on the left in the locality name (on right of heatmap) indicates the major drainage system (Amaz=Amazon, Arag=Araguaia, Pant=Pantanal and Para=Paraná. The middle of the notation gives a coded specific locality in the indicated drainage system (see Supplemental Table 1) and the last part of the notation is our archival number for the locality collection.

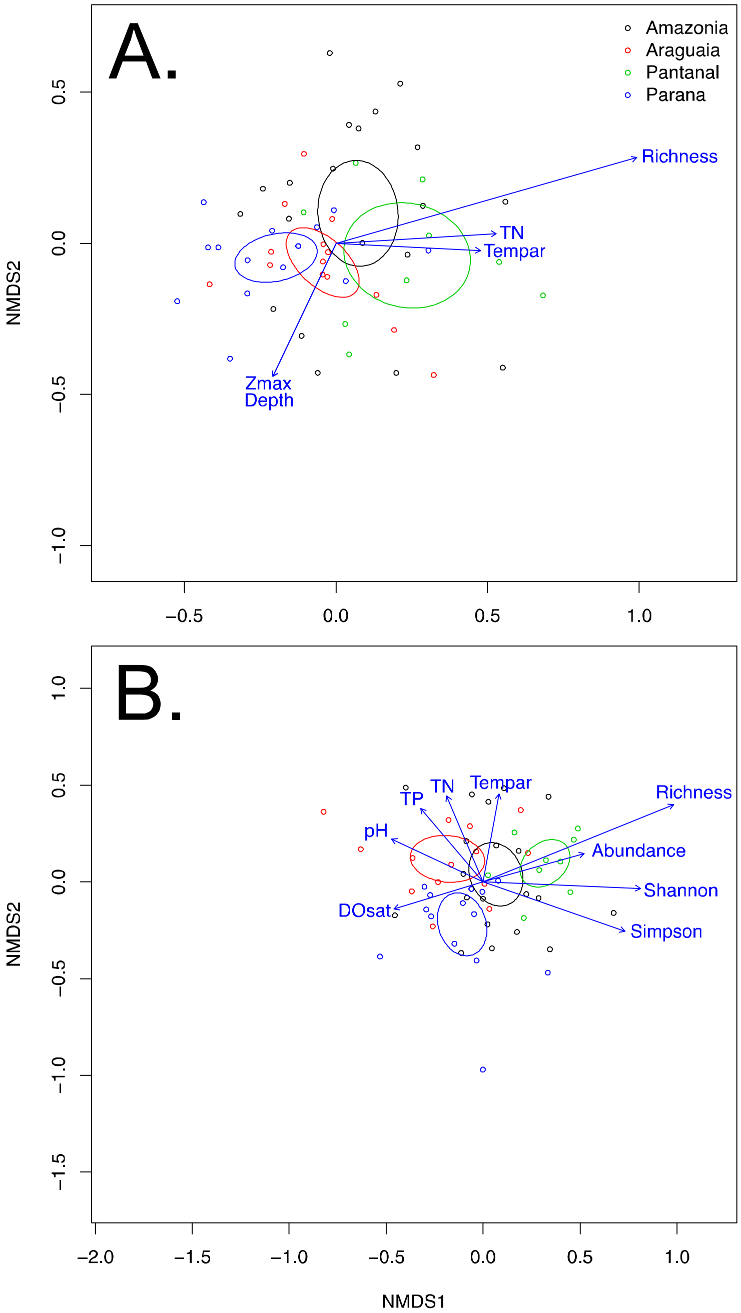

Supplemental Figure 9. NMDS ordinations of Brazilian floodplain lake sites using standard data of bacterioplankton identified to the phylum (A.) and family (B.) levels, highlighting the drainage system where a sample was located. Ellipses are for standard errors. Vectors represent environmental variables that significantly correlated with the ordination space, with length corresponding to the strength of the correlation.
